# Recent Progress on Polyphenols of Malaysian Honey and Their Molecular Mechanism Pathways in Cancer—A Comprehensive Review

**DOI:** 10.3390/ijms27073074

**Published:** 2026-03-27

**Authors:** Mohd Hayrie Mohd Hatta, Nazirah Amran, Farah Hidayah Kamisan, Maryam Hannah Daud, Mariatul Farhana Abdul Manaf, Kanaga Kumari Chelilah, Norwahidah Abdul Karim

**Affiliations:** 1 Centre for Research and Development, Asia Metropolitan University, Masai 81750, Johor, Malaysia; hayrie@amu.edu.my; 2 Department of Science Programmes, Sunway College Johor Bahru, Jalan Austin Heights Utama, Taman Mount Austin, Johor Bahru 81100, Johor, Malaysia; naziraha@sunway.edu.my; 3 Faculty of Health Sciences, Asia Metropolitan University, Masai 81750, Johor, Malaysia; farah.hidayah@amu.edu.my (F.H.K.); maryam@amu.edu.my (M.H.D.); kanaga@amu.edu.my (K.K.C.); 4 School of Healthcare Management, KPJ Healthcare University, Nilai 71800, Negeri Sembilan, Malaysia; mariatul.farhana@kpju.edu.my; 5 Department of Biochemistry, Faculty of Medicine, Universiti Kebangsaan Malaysia, Kuala Lumpur 56000, Malaysia

**Keywords:** anticancer activities, apoptosis, cancer cell proliferation, honey, mitochondrial pathway, polyphenol compounds

## Abstract

Cancer ranks as one of the top causes of death worldwide, and the World Health Organisation (WHO) estimates an increase of up to 55% in cases over the next 15 years, reaching 300 million cases worldwide. Current approaches to the treatment of cancer, such as chemotherapy and radiation therapy, have been used with continuous significant advancements. However, these conventional methods have harmful side effects that can last a lifetime. Today, there is growing interest in developing alternative cancer therapies from natural products or complementary medicine. One of the natural sources that has shown promise as an anticancer agent is honey, which has long been applied as a complementary medicine, and its beneficial health effects on various diseases in both animal and human models have been widely studied. Malaysian honey, such as Tualang, pineapple, Gelam, Kelulut, and Acacia, possesses a rich composition of phytochemicals, including polyphenols and flavonoids, which are reported to have promising anticancer properties. Examples of the phytochemicals highlighted in this review are phenolic acid, syringic acid, salicylic acid, p-coumaric acid, gallic acid, benzoic acid, caffeic acid, chrysin and its derivatives, kaempferol, fisetin, catechin, apigenin, quercetin, acacetin, pinocembrin, pinobanksin, hesperetin, naringenin, vitexin, isoorientin, xanthohumol, and galangin. This review highlights the anticancer mechanisms and molecular pathways of the phytochemicals found in Malaysian honey, focusing on their antioxidant effects, induction of mitochondrial-mediated apoptosis, inhibition of angiogenesis and metastasis, and suppression of cancer cell proliferation. The findings of various studies published in the past five years are collated to understand their mechanisms of action.

## 1. Introduction

Cancer can be defined as unregulated cell growth capable of invading or spreading to other regions of the body [1]. Today, cancer is a leading cause of global mortality and one of the most widespread illnesses, with approximately 9.7 million deaths and nearly 20 million new cases reported worldwide in 2022. Among cancer types, lung cancer is the leading cause of cancer-related mortality, followed by colorectal, liver, breast, stomach, and pancreatic cancers [2]. In Malaysia, a total of 48,639 cancer cases were reported in 2020, with this number predicted to double by 2040. Therefore, strategies to reduce the incidence of cancer worldwide are urgently needed to lower risk and improve the lives of those affected by the disease [3]. Generally, in cancer, the uncontrolled process of transforming, growing, and spreading abnormal cells is called carcinogenesis, and understanding this process is crucial for developing strategies for cancer prevention, early detection, and effective treatment [4].

**Figure 1:**
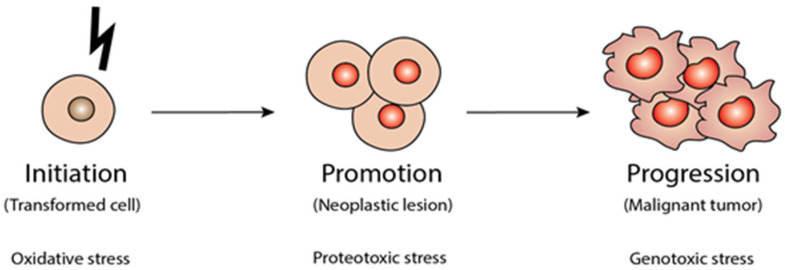
Illustration of the three phases in carcinogenesis. In the beginning, oxidative stress and reactive oxygen species (ROS) damage cell membranes, proteins, and DNA. During the promotion stage, cancer cells accumulate additional DNA mutations and produce abnormal proteins, which cause cellular stress. In the final stage, cancer cells become more aggressive due to unstable chromosomes and further DNA damage. This figure is reproduced from Ferreira et al. (2019) [6], an open access article published by MDPI (Basel, Switzerland) under a Creative Commons Attribution license 4.0 (CC-BY 4.0).

Carcinogenesis typically occurs in three stages, which are initiation, promotion, and progression. The first stage of cancer is called initiation, where an external factor, such as the presence of carcinogens, leads to irreversible genetic damage. Meanwhile, promotion involves the clonal expansion of altered cells, during which initiated cells are stimulated to divide and proliferate, potentially driven by factors such as hormones, inflammation, or specific chemicals. The final stage, progression, occurs when tumour cells acquire additional genetic and epigenetic changes, leading to increased malignancy, invasiveness, and a greater potential for metastasis [5]. Therefore, understanding these molecular mechanisms across the phases of carcinogenesis is essential for developing effective cancer therapies. Figure 1 illustrates the three-phase process of carcinogenesis following exposure to a carcinogen.

**Figure 2:**
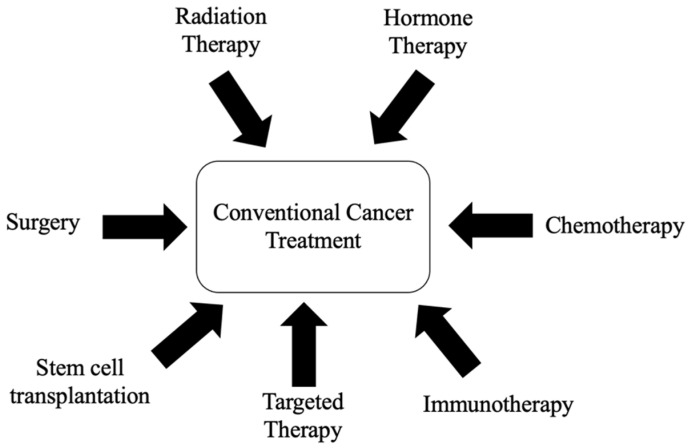
Examples of the conventional cancer treatments [12].

Conventional cancer treatments and therapies include surgery, chemotherapy, radiotherapy, targeted therapy, stem cell transplantation, hormone therapy, and many others, as shown in Figure 2 [7]. However, conventional cancer treatments are often associated with numerous limitations, such as postoperative recurrence, drug resistance, and toxicity [8,9]. For example, chemotherapy drugs can kill cancerous cells; however, they can also damage normal and healthy cells during treatment [10]. On the other hand, radiation therapy, which is highly effective in treating early-stage cancer, utilises a high dose of radiation to destroy cancer cells and shrink tumours. However, similar to chemotherapy, the main drawback is that radiation also deposits doses in healthy tissue before reaching its target [11]. While stem cell transplantation offers regenerative and therapeutic potential, this approach faces significant challenges in cell delivery, tracking, and immune responses [12]. All these challenges in conventional cancer therapeutics have led to a significant interest in complementary medicine and natural products for their chemopreventive and therapeutic potential.

In comparison to conventional cancer therapies, complementary medicine or alternative medicine could offer various health benefits, such as fewer side effects, greater availability, and more affordable treatment prices [13]. In general, the complementary or alternative medicines associated with cancer therapy include herbs, acupuncture, homoeopathy, and naturopathy, to name a few [14]. However, many of these approaches and therapies still lack reliable evidence of effectiveness before proceeding to clinical trials. Moreover, many complementary or alternative treatments have unproven risks and potential benefits [15].

Among the growing products with potential uses in complementary medicine for cancer treatment is honey, a natural substance made by honeybees from nectar [16]. For the past few years, honey has become the most investigated natural product for its anticancer effects, owing to its high polyphenol content and relative non-toxicity. Natural honey contains over 200 compounds, consisting mainly of sugar, water, enzymes, vitamins, minerals, volatile organic compounds, pigments, and especially phenolic compounds [17]. Besides that, honey-based products have been used for over a century as a food resource and for medical purposes [18]. Despite its mixture of sugars, the abundance of chemical compounds in honeys has long been recognised for its medicinal benefits, suggesting that these compounds can override the potential pro-carcinogenic effects of sugars. Recent studies strongly indicate that honey contains bioactive compounds that may be beneficial in cancer therapy. For instance, reactive oxygen species (ROS) and inflammation play important roles in carcinogenesis [19], and the antioxidant and anti-inflammatory properties of honey have been well documented in various studies [20,21,22]. These effects are largely attributed to its phenolic compounds [15,19,20], which help counteract the pro-carcinogenic effects of sugars and contribute to honey’s overall anticancer activity.

Among the phenolic compounds associated with anticancer properties are caffeic acid and its ester derivatives, gallic acid, p-coumaric acid, salicylic acid, trans-cinnamic acid, syringic acid, benzoic acid, flavonoid derivatives, chrysin and its derivatives, kaempferol, fisetin, catechin, apigenin, quercetin, acacetin, pinocembrin and pinobanksin, hesperetin, naringenin, vitexin, isoorientin, xanthohumol, and galangin, all of which will be discussed throughout this review. It has been reported that the phenolic content of honey can vary depending on its floral source, geographical origin, and the analytical methods applied in the laboratory [21]. Nonetheless, honey contains constituents that may offer anticancer properties, although the effectiveness is likely to vary with its composition [22]. The properties of honey and its therapeutic potential are discussed in the next section.

While it is acknowledged that there are numerous reviews and research studies, both past and present, regarding honey and its potential anticancer applications, this review comprehensively highlights research from the past five years (2021–2024) on the molecular mechanisms of phytochemicals found in Malaysian honey varieties such as Tualang, Kelulut, Acacia, and Gelam. It focuses on their effects against various common cancers (e.g., breast, lung, colon) studied through in vivo, in vitro, and clinical approaches, which, to our knowledge, have not been covered in previous literature. Moreover, this review highlights detailed studies on the anticancer properties of phenolic compounds in Malaysian honey, including polyphenols and flavonoid acids, with particular emphasis on their derivatives, such as chrysin, fisetin, catechin, apigenin, hesperetin, quercetin, gallic acid, salicylic acid, and others. The molecular mechanisms and pathways of each derivative are explored to highlight their effectiveness, enhance understanding, and provide suggestions for further studies. This review also aims to present these findings to health authorities as evidence of honey as a promising anticancer agent, warranting more in-depth studies before progressing to clinical trials.

### 1.1. Therapeutic Potential of Honey

Honey, a natural sweet substance produced by honeybees from plant nectar or secretions, has been valued across different cultures for its nutritional, medicinal, and health-promoting properties since ancient times [23]. Evidence of honey consumption dates back 8000 years, as depicted in Stone Age paintings, and it has been mentioned in Sumerian clay tablets (ca. 6200 BC), Egyptian papyri, Vedic texts, the Bible, and Hippocratic writings [24]. The medicinal properties of honey are also highlighted in the Holy Quran, where it is acclaimed for its healing qualities [25]. Ibn Sina, an ancient Persian physician, considered honey one of the best treatments for tuberculosis [26]. Honey has been used for wound healing throughout history, including during World War I, when it was employed to treat infected wounds and accelerate healing [27]. Meanwhile, in China, honey has traditionally been applied to inflamed wounds to reduce pain [28]. Hence, the medicinal properties of honey have been recognised for centuries, owing to its healing effects.

To date, honey has been used in various kinds of medicinal treatments, such as for asthma, eczema, fatigue, hepatitis, throat infections, tuberculosis, ulcers, and wounds, as well as a nutritious supplement [24]. Studies have shown that honey possesses remarkable antibacterial properties against human pathogenic bacteria such as *Escherichia coli* (*E. coli*) and *Pseudomonas aeruginosa* (*P. aeruginosa*) [29]. Moreover, studies have shown that honey possesses significant antioxidant activity, and the amount of antioxidants can be influenced by the plants selected by bees [30]. Recent studies have shown that honey-fried licorice protects against arrhythmia while alleviating associated oxidative stress and tissue damage [31]. In one case–control study, medical-grade honey was reported to positively affect the healing process of non-healing wounds in five patients with diabetic foot syndrome [32]. All these studies demonstrate the therapeutic potential of honey and suggest it could be useful for treating various diseases.

### 1.2. Composition of Honey and Its Anticancer Properties

Honey consists of over 200 components, mostly sugars (80–85%) and water (15–21%) [17,33,34]. It also contains proteins, enzymes (invertase, glucose oxidase, catalase, phosphatases), amino acids, organic acids (gluconic acid, acetic acid), lipids, vitamins (ascorbic acid, niacin, pyridoxine, thiamine, riboflavin, nicotinic acid, pantothenic acid), volatile chemicals, polyphenols (phenolic acids, flavonoids), carotenoids, and minerals [35,36,37,38,39,40]. Honey is rich in various phytochemical compounds, including vanillic acid, syringic acid, luteolin, apigenin, and others, as shown in Appendix A, which are known to exhibit potent antioxidant activities [41,42,43,44,45,46]. Despite the presence of various phytochemical compounds in honey, these compounds vary depending on the floral sources, geographical origin, entomological sources, season, environment, and processing factor [37,42,47,48,49].

**Figure 3:**
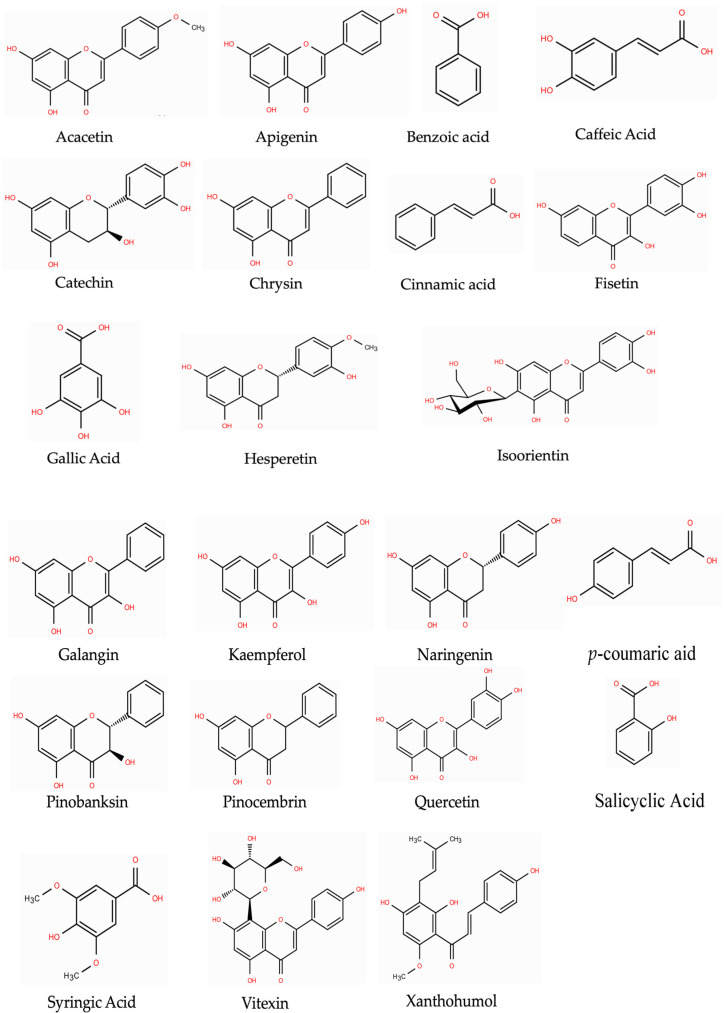
Examples of molecular structures of polyphenols found in Malaysian honey.

In Malaysia, five main types of honey are commonly consumed for their health benefits: Acacia, Gelam, Pineapple, Kelulut, and Tualang honey (TH). These honeys have been scientifically studied for their anticancer activities through various experiments, including clinical studies. A summary of past anticancer studies on Malaysian honey is provided in Appendix B. It is suggested that the anticancer properties of these honeys could be attributed to the presence of polyphenolic compounds such as quercetin, gallic acid, caffeic acid, and rosmarinic acid, among others, which have been reported to possess anticancer effects. Figure 3 shows the molecular structures of the polyphenols found in Malaysian honey and discussed in this review. Thus, in this review, we highlighted the pathway underlying the anticancer properties of the most abundant polyphenols found in Malaysian honey. We also summarise recent evidence ranging from laboratory experiments to clinical studies to provide readers with a clearer understanding of how these polyphenols modulate key pathways involved in cancer progression and their potential therapeutic applications. In particular, this review focuses on elucidating the molecular mechanisms of these compounds at pharmacologically relevant concentrations achieved in preclinical models. Such mechanistic insights may guide the development of improved therapeutic strategies, including nanoparticle formulations, liposomal delivery systems, and synergistic combination approaches, which are currently being explored to overcome limitations in bioavailability and therapeutic efficacy.

## 2. Methodologies

A comprehensive literature review was conducted to compile current knowledge on the polyphenol content and anticancer activity of Malaysian honey, with a focus on Tualang (derived from Tualang tree, known as *Koompassia excelsa*), gelam (derived from gelam tree, known as *Melaleuca cajuputi*), pineapple (*Ananas comosus* (L.) *Merr*), kelulut (stingless bee honey), and acacia (*Robinia pseudoacacia*) honey. These honeys were selected because of the abundance of research documenting their anticancer effects in whole form, whether processed or raw, although individual bioactive compounds have not been systematically summarised. Relevant articles were retrieved from PubMed/Medline, Scopus, ScienceDirect, and Google Scholar, covering publications from the last five years and all available records up to 30 June 2024. Search terms included combinations of Tualang, gelam, pineapple, kelulut, acacia, honey, polyphenols, flavonoids, phenolic acids, cancer, molecular mechanism, and signalling pathways. Boolean operators (OR, AND, NOT) were applied to optimise search precision and retrieve relevant records. Studies reporting on polyphenols identified in these Malaysian honeys, their anticancer activities as individual compounds, and the molecular signalling pathways and mechanisms underlying these polyphenols’ anticancer effects were selected and summarised in tables and figures. Although many studies examined individual compounds in non-Malaysia honey or other natural sources, these data were integrated to support the argument that the polyphenols present in Malaysian honey contribute to its potent anticancer activity. This present paper highlights how these compounds influence key signalling pathways involved in cancer, providing a scientific rationale for the development of natural product-based anticancer agents.

## 3. Molecular Mechanism of Polyphenols in Modulating Key Pathway of Cancer Progression

### 3.1. Effect of Phenolic Acid in Cancer

#### 3.1.1. Hydroxycinnamic Acids

##### Caffeic Acid and Its Esters

Caffeic acid is a hydroxycinnamic acid (C6–C3 structure) with two OH groups and a carboxylic acid on its side chain. Its aromatic ring and conjugated double bond easily trap free radicals by donating hydrogen and delocalizing electrons. Additionally, the para-OH group enhances this stability. Caffeic acid also chelates metal ions with its two OH groups to prevent peroxide breakdown into harmful radicals, acting as both a primary and secondary antioxidant [50,51]. To date, numerous positive effects of caffeine on human health have been demonstrated. For example, it primarily inhibits the PI3K/Akt, MAPK, and NF-κB signalling pathways, which, in turn, suppress vascular endothelial growth factor (VEGF) expression, thereby limiting cancer cell proliferation. Caffeic acid has also been shown to improve redox status, normalise hepatic enzyme levels, reduce NF-B signalling, decrease advanced glycation end-products signalling, and decrease blood glucose levels in diabetic animals [50]. Due to its pro-oxidant and antioxidant properties, a diet rich in caffeic acid have been demonstrated to suppress cancer development. Regarding oxidative deoxyribonucleic acid (DNA) damage and subsequent signalling, caffeic acid exhibits pro-oxidative effects in cancer cells, leading to the induction of apoptosis (programmed cell death) [52]. Furthermore, it is reported that heterocyclic amines, which can cause cancer and are mutagens such as PhIP (2-amino-1-methyl-6-phenyl-imidazo[4,5-b]pyridine) found in hot foods high in protein, can be inhibited by caffeine [53].

Yang and colleagues reported that caffeic acid at approximately 20 µM concentration downregulated the expression of mortalin (mitochondrial 70 kDa heat shock protein), which acts as an upstream activator of the PI3Kβ, NF-κB, and VEGF signalling pathways. Three hepatic cell lines, including HepG2, Hep3B, and sorafenib-resistant HuH7 cells, were used to observe these effects. It was observed that a higher concentration of caffeine (1 mM) inhibited the growth of hepatocellular carcinoma WCH-17A cells and induced apoptosis by interfering with mitochondrial function [54]. Additionally, it reduced the expression of cyclin D, beta-catenin, phospho-MEK1/2, phospho-ERK1/2, and vimentin, which are markers of the epithelial–mesenchymal transition and promoters of cell proliferation [55]. The exposure to 100 µM caffeic acid activated AMP-kinase (AMPK), a metabolic sensor with anti-tumour properties, in the aggressive metastatic human cervical HTB-34 (ATCC-CRL1550) cancer cell line [56]. Meanwhile, in human Simpson-Golabi-Behmel syndrome (SGB) adipocytes, caffeine with various concentrations (5 µM, 10 µM, and 50 µM) increased the expression of brown adipocyte markers, such as cell death activator CIDE-A (CIDEA) and uncoupling protein 1 (UCP1), and decreased the expression of key genes of white adipogenic differentiation, such as adiponectin, CAAT/enhancer-binding protein alpha (CEBPA), and fatty acid-binding protein 4 (FABP4) [57].

Propolis contains a naturally derived substance called caffeic acid phenethyl ester (CAPE). This medicinal plant is well-known for its profound therapeutic effects, which include its potency as an anti-diabetic, hepatoprotective, and neuroprotective agent [58]. CAPE’s ability to suppress tumours has attracted significant attention among scientists, with both in vivo and in vitro studies supporting this finding. Caffeic acid may induce apoptosis in cancer cells by increasing ROS levels and compromising mitochondrial function [59]. In cancer treatment, caffeic acid and its derivatives affect molecular pathways, including AMPK and PI3K/Akt, that promote cancer progression. By inhibiting the epithelial–mesenchymal transition, caffeic acid suppresses metastasis and reduces the aggressive behaviour of malignancies [60,61]. Notably, caffeic acid and CAPE could increase cancer cells’ sensitivity to chemotherapy-induced cell death and enhance their responsiveness to the drug. Caffeic acid and CAPE have been co-administered with other anti-tumour chemicals, such as gallic acid and p-coumaric acid, to enhance their ability to suppress cancer [59,60]. In addition, nanocarriers have been introduced to improve their potential to control cancer, given their low bioavailability [62]. In the current study, these topics are covered with an emphasis on the molecular pathways that enable the rapid conversion of caffeic acid for therapeutic application [60]. CAPE has been shown to inhibit breast cancer cell migration, invasion, and the epithelial–mesenchymal transition (EMT). Mechanistically, CAPE prevents nuclear transfer and fibroblast growth factor receptor 1 (FGFR1) phosphorylation, whereas FGFR1 overexpression attenuates CAPE’s anti-metastasis effect. Furthermore, it was shown that FGFR1 is coupled to MD2 and that FGFR1 phosphorylation, nuclear translocation, and cell invasion are inhibited by silencing MD2 [63].

**Figure 4:**
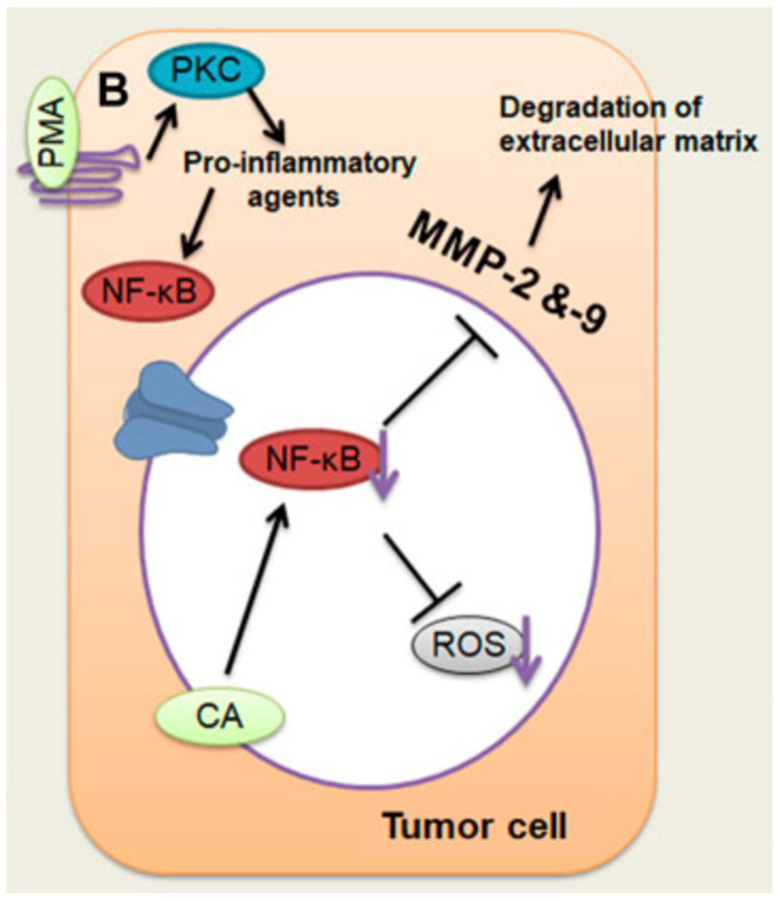
Caffeic acid may inhibit tumour cell invasiveness and growth by inhibition of MMP-2 and MMP-9 expression, which subsequently blocks PMA-induced activation of NF-κB (activating protein 1) in tumour cells (↓). This figure is reproduced from Alam et al., (2022) [61], an open access article published by Frontiers Media S.A under Creative Commons Attribution License 4.0 (CC BY 4.0).

Caffeic acid plays a significant role in inhibiting cancer growth by inducing apoptosis and reducing cell viability [60]. Treatment with caffeic acid alters the cell cycle, halts cancer progression, and modulates caspase expression. Specifically, caffeic acid induces apoptosis by inhibiting Bcl-2 activity, releasing cytochrome c (cyt-c), and subsequently activating caspase-3, thereby triggering apoptosis via the intrinsic apoptotic pathway. Moreover, the caffeic acid has been shown to be effective against a variety of malignancies by reducing vascular endothelial growth factor (VEGF)-mediated vascularisation, suppressing matrix metalloproteinase-2 (MMP-2) and MMP-9, and preventing the overproduction of ROS, which in turn promotes the killing of cancer cells through DNA oxidation and angiogenesis. The inhibitory effect of caffeic acid on MMP-2 and MMP-9 is linked to the suppression of NF-κB activation, as demonstrated in phorbol 12-myristate 13-acetate (PMA)-induced cancer cells, which diminishes the invasiveness and proliferation of cancer [61]. Figure 4 shows that caffeic acid reduces MMP-2 and MMP-9 by blocking NF-κB activation in liver tumour cells stimulated with PMA, thereby lowering cancer invasiveness and growth.

#### 3.1.2. Gallic Acid

##### *p*-Coumaric Acid

**Figure 5:**
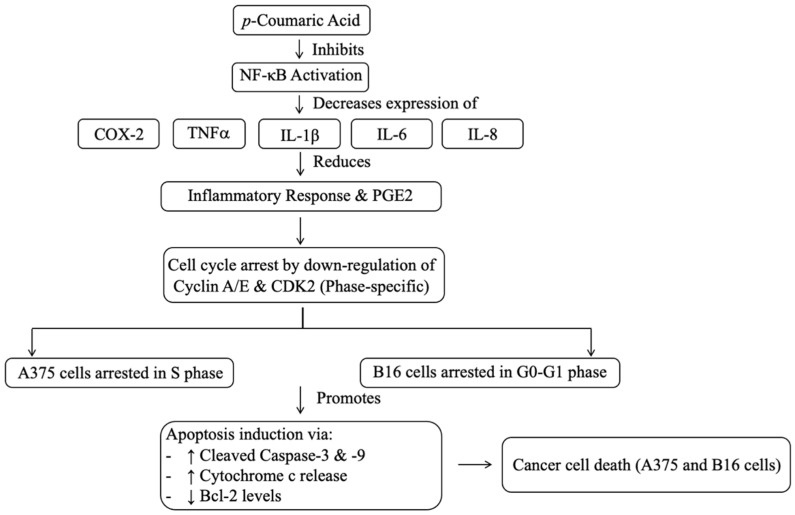
Molecular mechanism of *p*-coumaric acid-induced apoptosis and anti-inflammatory effects in A375 and B16 cancer cells.

*p*-Coumaric acid, a hydroxycinnamic acid (C_9_H_8_O_3_), features an aromatic ring with one OH group linked to an unsaturated C3 side chain with a carboxylic acid. It is the most common isomer found in plants, mushrooms, vegetables (tomatoes, carrots), fruits (grapes, apples), grains, and beverages (tea, coffee, wine). The conjugated structure enables strong antioxidant activity by trapping free radicals [64]. Studies have shown that *p*-coumaric acid significantly suppresses NF-κB activation and expression, as well as the expression, synthesis, and secretion of several inflammatory response mediators, including COX-2, TNFα, IL-1β, IL-6, IL-8, and prostaglandin E2 (PGE2). The enzyme COX-2 is specifically overexpressed in colon tumours and is involved in the production of PGE2 from arachidonic acid, which triggers inflammatory reactions. Therefore, pharmacological suppression of COX-2 may reduce the incidence of colorectal cancer [64]. Meanwhile, the proliferation of human melanoma A375 cells and mouse melanoma B16 cells is strongly and dose-dependently inhibited by *p*-coumaric acid, which also induces notable changes in cell morphology. Additionally, *p*-coumaric acid downregulates the cell cycle-related proteins Cyclin A and CDK2, causing S phase arrest in A375 cells, while downregulating Cyclin E and CDK2 to induce G0–G1 phase arrest in B16 cells. Furthermore, *p*-coumaric acid significantly promotes the death of both B16 and A375 cells. The *p*-coumaric acid also increases the levels of cleaved caspase-3, cleaved caspase-9, and cytoplasmic cytochrome c (Cyto-c), while downregulating Bcl-2 expression. These molecular changes ultimately induce apoptosis in A375 and B16 cells [65]. Figure 5 illustrates the molecular pathway of *p*-coumaric acid-induced apoptosis and anti-inflammatory effects in cancer cells.

#### 3.1.3. Hydrobenzoic Acids

##### Gallic Acid

Gallic acid (C_7_H_6_O_5_), a trihydroxybenzoic acid, has been reported to exhibit strong antioxidant activity through its three phenolic OH groups that donate hydrogen to neutralize free radicals and chelate pro-oxidant metals [66]. As a potential anticancer agent, apoptosis- and breast cancer-related proteins can interact antagonistically with several proteins associated with gallic acid and its derivatives, such as N-alkyl gallamide. Among these, the proteins JUN, AKT1, CASP3, and CASP7 exhibited the highest confidence scores. Gallic acid derivatives, such as N-octyl gallamide, N-tert-butyl gallamide, and N-isoamyl gallamide, have shown strong interactions with these proteins via molecular docking, suggesting that these derivatives may possess significant biological activity. According to in vitro studies by Arsianti et al. (2024) [67], these gallic acid derivatives can induce apoptosis, potentially reducing the proliferation of MCF7 breast cancer cells by 50%. Meanwhile, studies conducted in vitro and in vivo showed that gallic acid dose-dependently significantly reduced the invasion, migration, and proliferation of clear cell renal cell carcinoma (ccRCC) cells. Hence, this finding showed that gallic acid significantly induced the release of markers associated with autophagy, which in turn regulated the PI3K/Akt/Atg16L1 signalling cascade [68].

##### Salicylic Acid

Salicylic acid (C_7_H_6_O_3_), also known as 2-hydroxybenzoic acid, is a simple phenolic acid with an OH group next to a COOH group on a benzene ring. This ortho positioning creates intramolecular hydrogen bonding, making it less soluble in water yet highly bioactive [69]. It can be found in plants such as willow bark and spices; its phenolic OH group enables antioxidant activity by scavenging free radicals [69]. Both salicylic acid and its derivative, acetylsalicylic acid (aspirin), are traditional medications with a variety of medical uses. Ausina et al. [70] demonstrated that both salicylic acid and aspirin exhibited anticancer effects in an implanted melanoma murine model. The pro-apoptotic effects of these compounds were also confirmed in melanoma B16F10 cells cultured in both 3D and 2D conditions. The in vitro and in vivo studies showed that both salicylic acid and aspirin induced endoplasmic reticulum (ER) stress, resulting in the overexpression of the pro-apoptotic transcription factor C/EBP homologous protein (CHOP). Meanwhile, in a p53-independent manner, salicylate stimulates the production of tumour-suppressive microRNAs encoded by the miR-34a and miR-34b/c genes. Salicylate activates AMPK, which in turn activates NRF2; through ARE motifs, NRF2 directly promotes the expression of miR-34a, miR-34b, and miR-34c. Furthermore, by activating AMPK, salicylate inhibits c-MYC, a known regulator of NRF2-mediated transactivation. Salicylate-induced suppression of c-MYC is required for NRF2-mediated activation of miR-34a/b/c. The inhibitory effects of salicylate on colorectal cancer (CRC) cell motility, invasion, and metastasis were largely abolished by inactivation of miR-34a/b/c [71].

In human colorectal cancer cells treated with salicylic acid, silencing of ribosomal protein S3 (RPS3) caused G1-phase arrest and decreased cyclin-dependent kinase 4 (CDK4) expression [72]. Thus, one possible mechanism by which salicylic acid protects against colorectal cancer is via its adverse effect on RPS3 function. In addition, salicylic acid inhibits tumour growth by activating AMP-activated protein kinase (AMPK). The AMPK is triggered either by allosteric effects of adenosine monophosphate/adenosine diphosphate (AMP/ADP) on its subunits or by phosphorylation of its α and β subunits by LKB1 and calmodulin-dependent protein kinase-kinase (CAMKK). Furthermore, salicylic acid can directly bind to and activate AMPK. Once activated, AMPK phosphorylates and inactivates acetyl-CoA carboxylase (ACC). The inactivation of ACC subsequently inhibits tumour cell proliferation and fatty acid synthesis [73].

##### Syringic Acid

Syringic acid (3,5-Dimethoxy-4-hydroxybenzoic acid) is one of the abundant phenolic compounds found in olives, dates, spices, pumpkin, grapes, honey, red wine, and other plants. Due to its excellent pharmacological properties, it exhibits various health benefits, including antioxidant, antimicrobial, anti-inflammatory, anti-diabetic, and anticancer effects [74]. As a polyphenol derivative, syringic acid is emerging as a modulator of diverse transcriptional factors and an activator of apoptosis-related proteins in cancer cells. This makes syringic acid a potential candidate for an anticancer agent in the future [75]. In general, the anticancer effects of phytochemicals are influenced by their capacity to neutralise free radicals and stimulate antioxidant enzymes. Moreover, elevated levels of ROS can cause alterations in double-stranded DNA, potentially leading to the formation of a neoplasm [76]. Syringic acid and its derivatives exhibit anticancer effects through multiple mechanisms. These include downregulating cell proliferation markers such as PCNA, Cyclin D1, and mutant p53, as well as cell cycle proteins CDK4, CDK6, and cyclins B, C, and H. Additionally, they upregulate apoptotic genes, such as Bax, Caspase 2, 3, and 9. Syringic acid also exhibits anti-mitogenic and chemo-sensitising effects by disrupting cell migration, NF-κB-DNA binding, apoptosis regulation, and inducing cell cycle arrest [77]. In this section, the anticancer activity of syringe acid against various cancer cell lines and its molecular mechanisms are comprehensively reviewed.

The in vitro effects of syringic acid on colorectal cancer cells (SW-480) and the effects of orally administered syringic acid on in vivo models of colorectal cancer induced in rats by 1,2-dimethylhydrazine (DMH) were evaluated by Mihanfar et al. [78]. They found that syringic acid exerted potent anti-proliferative and pro-apoptotic effects in SW-480 colorectal cancer cells, with its anticancer effects mediated by inducing oxidative DNA damage. Moreover, syringic acid modulated the expression of proliferative genes, including p53, ERK1/2, AKT, PI3K, and NF-κB, in SW-480 cells. Additionally, syringic acid activated autophagy in SW-480 cells. Consequently, it can be concluded that oral administration of syringic acid in rats with DMH-induced colorectal cancer significantly suppressed tumour progression [78]. Meanwhile, previous studies have shown that syringic acid exerts chemotherapeutic and chemosensitising effects in human colorectal cancer cells through various mechanisms, including cell-cycle arrest, induction of apoptosis, inhibition of proliferation, migration, and angiogenesis, NF-κB DNA-binding, and proteasome activity [79]. These results suggest the potential of syringic acid as an anticancer agent against human colorectal cancer cells, particularly due to its ability to regulate diverse pathways associated with tumour growth and spread.

Studies have also shown that syringic acid exhibits anticancer activity by decreasing mitochondrial membrane potential (MMP) and cell viability while increasing intracellular ROS levels. In gastric cancer cells, syringic acid selectively induced apoptosis in a dose-dependent manner by enhancing the activation of caspase-3, caspase-9, and poly ADP-ribose polymerase (PARP), while reducing the expression of p53 and BCL-2. It also reduced the activities of antioxidant enzymes such as superoxide dismutase (SOD), catalase (CAT), and glutathione peroxidase (GSH-Px), while increasing thiobarbituric acid-reactive substances (TBARS). Furthermore, syringic acid inhibited gastric cancer cell proliferation and inflammation and promoted apoptosis by upregulating mTOR via the AKT signalling pathway. That said, syringic acid suppresses gastric cancer by inducing apoptosis, reducing inflammation, and modulating the mTOR/AKT pathway [80].

Although several studies have demonstrated the potential of syringic acid for treating lung cancer cells, its therapeutic effects could be enhanced by modification or by combining it with zinc oxide (ZnO). In lung cancer models using A549 cells, ZnO-loaded syringic acid significantly improved body weight and reduced serum marker enzyme levels compared to BAP-treated animals. Cytokine analysis also showed increased levels of TNF-α, IL-6, and IL-1β in ZnO-syringic acid-treated mice compared with BAP-induced lung cancer mice, as supported by histological findings. Furthermore, cytotoxicity assessments revealed changes in MMP, a profound increase in ROS and induction of apoptosis with ZnO-syringic acid treatment compared to controls. The study demonstrated that ZnO-syringic acid caused moderate, concentration-dependent ROS generation, disrupted mitochondrial membrane potential, and induced morphological changes, as confirmed by dual staining and cell adhesion assays evaluating cell viability [81].

Meanwhile, studies on the cytotoxicity of syringic acid against the HepG2 human hepatocellular carcinoma (HCC) cell line were conducted to assess its potential as an anticancer agent. The anticancer activity was evaluated by treating HepG2 cells with 25, 50, and 100 µM syringic acid for 24 h. Treatment with syringic acid resulted in significant cytotoxicity in HepG2 cells. Acridine orange AO and ethidium bromide staining revealed membrane blebbing and distortion in syringic acid-treated cells. The expression of apoptotic markers, including caspases 3 and 9, cytochrome c, Apaf-1, Bax, and p53, was increased upon the addition of syringic acid, suggesting the possibility of apoptosis induction in HepG2 cells. In addition, it resulted in significant downregulation of the Bcl-2 gene expression. Syringic acid exhibits a cytotoxic effect on the human HepG2 cell line, suggesting its potential as a promising agent in anticancer research [82].

The anticancer potential of syringic acid was further assessed using molecular docking simulations with the Gaussian 09 program. In silico studies demonstrated that syringic acid significantly inhibits breast cancer. Seven distinct breast cancer proteins, each associated with distinct mechanisms, were used to evaluate the docking efficiency of syringic acid. The results showed that syringic acid achieved the highest docking score with progesterone receptors (PR) and the human epidermal growth factor receptor-2 (HER-2) protein, at −7.7 kcal/mol. The high affinity for these proteins suggests potential therapeutic applications, as PR and HER-2 are key targets in breast cancer treatment due to their roles in tumour growth and progression [83]. Likewise, another in silico work by Cheemanapalli et al. [84] showed that both syringic acid and its derivative novel SA analogue (SA10) bind to the active site of NF-κB, thereby interfering with the association between DNA and NF-κB. The SA10 demonstrates a stronger binding affinity than syringic acid and remains stably formed within the interior of NF-κB, as supported by molecular mechanics Poisson–Boltzmann surface area (MM-PBSA) analysis. By suppressing NF-κB activation, it has the potential to delay the progression of cancers, inflammatory conditions, and other diseases by disrupting critical signalling pathways that regulate cell proliferation and survival. Exploring NF-κB inhibitors from phytocompound-based derivatives has recently gained significant attention in the search for therapeutic drugs against cancer, as these compounds may offer safer and more targeted alternatives to traditional treatments [84]. Therefore, it can be proposed that syringic acid has significant anticancer potential by inhibiting breast cancer through targeting PR and HER-2 proteins and by binding to NF-κB, potentially delaying cancer onset. It offers a promising therapeutic alternative with safer, more targeted effects than traditional treatments.

Besides those in silico studies, Cheemanapalli et al. [84] also evaluated the chemically synthesised novel SA10 for its anticancer activity using in vitro studies. A study using the K562 cell line showed that SA10 exhibited a higher inhibition rate (IC50 = 50.40 μg/mL) than syringic acid (IC50 = 96.92 μg/mL) at 50 μM. The inhibition ratio was determined by measuring NF-κB and Bcl-2 expression levels, revealing that SA10 exhibited a twofold greater inhibitory effect than syringic acid. This finding shows that SA10 functions as an NF-κB inhibitor and induces apoptosis, highlighting its significance given NF-κB’s crucial role in cancer cell survival and growth.

Recently, the use of pro-oxidants to counteract the effects of antioxidants by inducing oxidative stress could be developed as a novel approach in cancer studies. This approach exploits the vulnerability of cancer cells to oxidative damage, offering a potential therapeutic advantage by selectively targeting these cells while sparing healthy tissues [85,86]. Syringic acid is recognised as one of the natural compounds that exhibit antioxidant properties. Moreover, the addition of transition metal ions can influence oxidation processes, and elevated pH levels, along with high concentrations of phenolic compounds, can foster conditions that reveal their pro-oxidant activity. Therefore, using these approaches, Rashedinia et al. [87] investigated the effects of syringic acid on HEK 293 and HepG2 cells in the absence and presence of exogenous Cu (II) and Fe (II) ions. The studies revealed that exposure of HepG2 cells to syringic acid in the presence of Cu (II) for 72 h significantly decreased cell viability. Additionally, ROS production, apoptosis induction, and autophagic vacuole formation were significantly elevated in HepG2 cells, while mitochondrial membrane potential and mitochondrial mass remained largely unchanged. This suggests that the pro-oxidant activity of syringic acid and Cu (II) primarily targets cellular pathways involved in cell death and survival. Furthermore, syringic acid and Cu (II) significantly reduced the plating efficiency and survival fraction of cancer cells. Their combined treatment exerted cytotoxic effects on cancer cells and induced pro-oxidant activity. Moreover, this approach also triggered autophagy in cancer cells while exerting minimal cytotoxicity on normal cells, highlighting its potential as a promising candidate for novel cancer therapy development. The induction of autophagy is particularly significant as it can enhance the therapeutic efficacy by promoting cancer cell death while minimising harm to normal cells [87].

**Figure 6:**
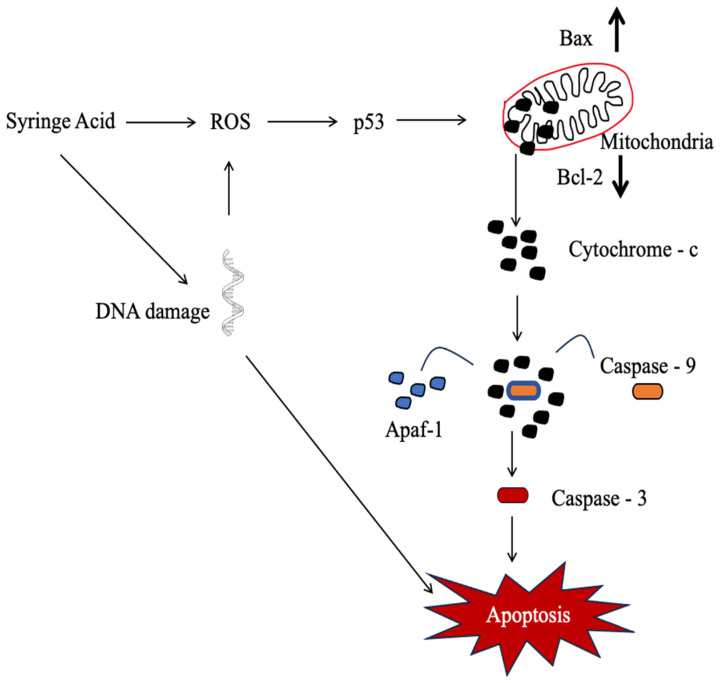
The proposed cytotoxic pathway of syringic acid in HepG2 cells. The cytotoxic effect of syringic acid on HepG2 cells is proposed to be mediated by ROS. Adapted from Gheena and Ezhilarasan (2019) [82].

To conclude, syringic acid demonstrated anticancer effects via multiple mechanisms across diverse cancer types. For example, it induces apoptosis by upregulating apoptotic proteins, such as caspases 3 and 9, and Bax, and downregulating anti-apoptotic proteins, such as Bcl-2. Syringic acid also modulates signalling pathways, including the mTOR/AKT pathway in gastric cancer, and suppresses cell proliferation by affecting cell cycle proteins. Additionally, it generates ROS, thereby contributing to its pro-oxidant activity and enhancing oxidative stress in cancer cells. Moreover, the potential of syringic acid to suppress NF-κB and modulate other cellular pathways further supports its development as an anticancer agent. Table 1 summarises the anticancer properties of syringic acid and its mechanism, while Figure 6 summarises the suggested cytotoxic pathway of syringic acid in HepG2 cells. The cytotoxic effect of syringic acid on HepG2 cells is proposed to be mediated by ROS.

### 3.2. Simple Aromatic Acids

#### 3.2.1. Trans-Cinnamic Acid

There is limited literature on trans-cinnamic acid; however, one study showed that cinnamic acid enhances the inhibitory effect of doxorubicin on the migration and invasion of cancer cells and increases the susceptibility of A549 cells to doxorubicin-induced apoptosis. The fact that cinnamic acid and doxorubicin exert significantly less effect on normal lung fibroblasts further emphasises the importance of these findings and supports the potential of cinnamic acid derivatives as safe chemosensitizers. According to these studies, cinnamic acid exhibits favourable in vitro ADME/T characteristics and can inhibit the CBR1 enzyme. This is evidenced by its ability to restrict the synthesis of doxorubicinol during doxorubicin biotransformation and by its preferential binding to the enzyme’s active site, as demonstrated through molecular modelling [88].

#### 3.2.2. Benzoic Acid

Studies have shown that when the anti-inflammatory aminobenzoic acid (DAB-1) was administered in vivo to ectopic and orthotopic cancers, it effectively reduced tumour growth, metastasis, and mortality. It also significantly inhibited tumour cell proliferation and reduced iNOS expression in tumour tissues. Furthermore, DAB-1’s anticancer effect was associated with the deactivation of macrophages in tumour-bearing animals. Mechanistic analyses demonstrated that DAB-1 effectively suppressed the activation of the TNFα/NFκB and IL6/STAT3 signalling pathways, as well as TNFα-induced NO production, by reducing NFκB transcriptional activity and functional iNOS expression. Furthermore, DAB-1 inhibited cell proliferation with negligible or no impact on apoptosis or cell death [89]. The expression levels of proteins of p-ERK, p-AKT, p-PI3K, and p-ER increased in a concentration-dependent manner. 4-Hydroxybenzoic acid is responsible for A. tegmentosum’s estrogen-like actions and may help regulate estrogenic effects during menopause, which are associated with breast cancer [90].

### 3.3. Effect of Flavonoids on Cancer

Flavonoids are naturally occurring phenolic phytoconstituents extracted from edible plants and honey, recognised for their diverse pharmacological properties [91]. Over the past decades, they have been extensively studied for their biological activities, particularly their anticancer effects [92]. Flavonoids are reported to play a crucial role in modulating cellular processes related to health and disease [93]. Moreover, they represent one of the major families of phenolic compounds identified in Malaysian honey [94]. Flavonoids are typically categorised into several subclasses, including flavanols (kaempferol, quercetin, and pinobanksin), flavones (chrysin, luteolin, and apigenin), flavanones (pinocembrin, naringenin, and hesperetin), isoflavones (genistein), and anthocyanidins [95].

Recent reports suggest that flavonoids from Malaysian honey possess potent anticancer properties. The molecular pathways underlying anticancer effects often involve modulating key signalling pathways, regulating gene expression, and interacting with specific cellular targets. One of the flavonoids identified in Malaysian honey is chrysin and its derivatives. Blue passion flowers (Passiflora caerulea), propolis, and honey are common sources of chrysin, a naturally occurring bioflavonoid [96]. Several studies have reported on the mechanisms of chrysin as a chemotherapeutic agent. According to Mohos et al. [97], chrysin glucuronide and chrysin sulfate are substrates for multidrug resistance-associated protein 2 (MRP2), a glutathione transporter. During this transport process, glutathione (GSH) is exported from cells by MRP2. Consequently, chrysin metabolites significantly increase intracellular GSH efflux, leading to GSH depletion and an accumulation of reactive oxygen species (ROS) within cancer cells [98]. Nonetheless, the extensive metabolism of chrysin, which limits its bioavailability, limits its potential use in cancer treatment [99].

#### 3.3.1. Chrysin and Its Derivatives

**Figure 7:**
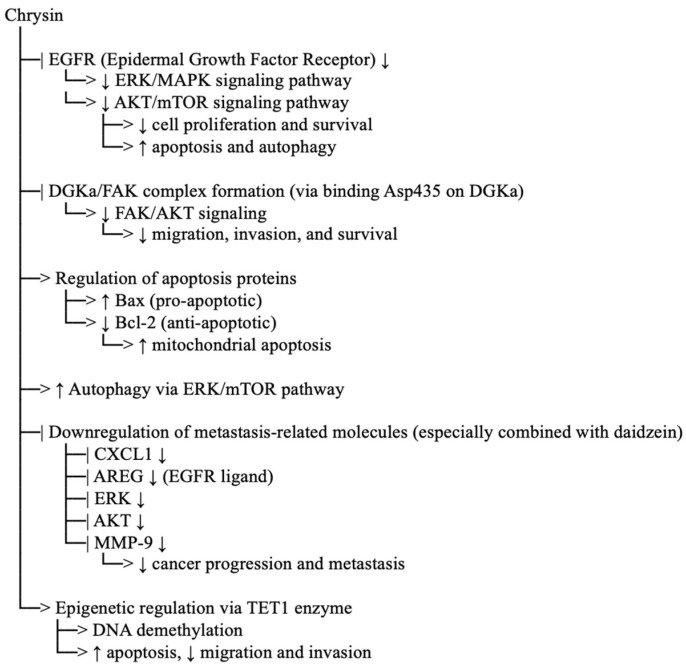
Molecular mechanisms of the pathway of chrysin in cancer cells. Chrysin demonstrated anticancer activity by inducing apoptosis via both mitochondrial and death receptor pathways. It activates pro-apoptotic proteins (Bax), blocks anti-apoptotic proteins (Bcl-2), and causes cell cycle arrest. Chrysin also increases oxidative stress (ROS), inhibits growth pathways such as PI3K/Akt, and regulates inflammatory pathways, including NF-κB and COX-2.

Chrysin, a 5,7-dihydroxyflavone, is a flavonoid with dual OH groups that enable potent antioxidant activity through radical scavenging and metal chelation [100]. In anticancer research, chrysin has been reported to inhibit the epidermal growth factor receptor (EGFR). Following a 48 h treatment with chrysin derivatives, the EGFR protein levels of MCF-7 cells intensely decrease dose-dependently [101]. Interestingly, Salama and Allam [102] also demonstrated that the combination of chrysin and daidzein has potential as a chemoprophylactic by downregulating CYP2E1. The combination also downregulates CXCL1, AREG, ERK, AKT, and MMP-9, which play vital roles in cancer progression and metastasis. In human gastric cancer cells (MGC-803), chrysin administration upregulates the pro-apoptotic gene Bax and downregulates the anti-apoptotic protein Bcl-2 in a dose-dependent manner [103]. Moreover, chrysin also induces apoptosis in the oesophageal squamous cell carcinoma (ESCC) cells, indicating its potential as a good anticancer agent [104]. In other studies, chrysin interfered with the formation of the DGKa/FAK complex by binding at the Asp435 site in the catalytic domain of DGKa, thereby inhibiting the FAK/AKT signalling pathway. Meanwhile, in MC-3 oral cancer cells, chrysin activates apoptosis by regulating the MAPK/extracellular signal-regulated kinase pathway and regulates ERK/mTOR-Mediated autophagy [105]. A Study demonstrated by Zhong et al. [106] also supported chrysin-induced apoptosis of the cancer cells. Regulation of the Ten-eleven translocation enzyme (TET1) by chrysin, which involves epigenetic modification, enhances cell apoptosis and inhibits cell migration and invasion in gastric carcinoma. Figure 7 shows the molecular pathway of chrysin in cancer cells.

#### 3.3.2. Kaempferol

Another important flavonoid present in honey is kaempferol. Kaempferol, a tetrahydroxyflavone, demonstrates strong antioxidant protection through its four strategically positioned phenolic hydroxyl groups [107]. Several studies in the literature have demonstrated the anticancer properties of kaempferol against various cancer cell lines, including breast cancer, head and neck cancer, cervical cancer, ovarian cancer, brain cancer, lung cancer, prostate cancer, and others [108]. It can be demonstrated that increased apoptosis in cancer cells is a key mechanism contributing to its anticancer effects. Kaempferol triggers apoptosis through several molecular pathways in cancer cells. Kaempferol induces apoptosis through multiple molecular pathways in cancer cells [109]. For example, kaempferol significantly suppresses the proliferation of triple-negative breast cancer (TNBC) MDA-MB-231 cells via an intrinsic mitochondria-dependent pathway, as evidenced by the activation of Caspases 9 and 3. The IC_50_ value of 43 μmol/L in this cell line is lower compared to that against the BT474 breast cancer cell line, which is positive for ER and HER2 receptors [110]. In another work, a conjugate of 1-deoxynojirimycin and kaempferol induced apoptosis via mitochondria-mediated pathways in MCF-7 cells. This derivative was shown to upregulate Bax expression and downregulate Bcl-2 expression, thereby regulating apoptosis [111].

**Figure 8:**
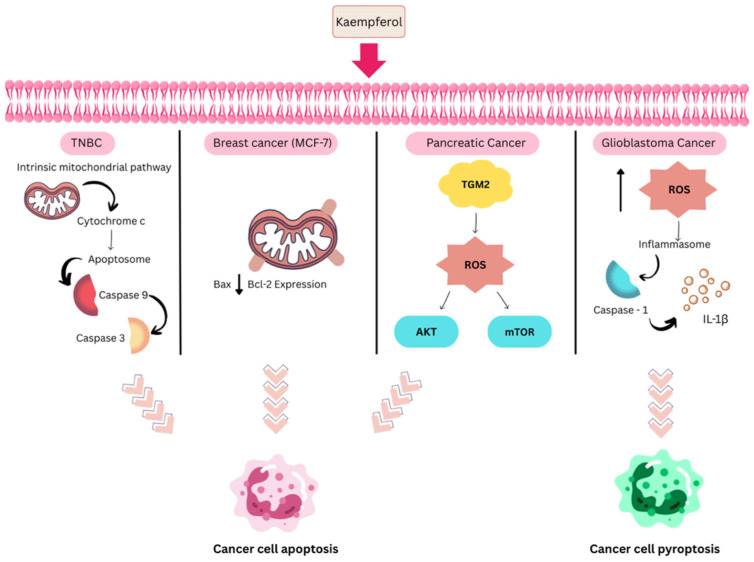
Molecular pathways of kaempferol as an anticancer agent in various cancer cell lines. The anticancer effects of kaempferol are mainly attributed to reducing proteins associated with cancer progression, inducing apoptosis, causing cell cycle arrest, and lowering levels of anti-inflammatory proteins.

In other studies, Wang et al. [112] reported that kaempferol exhibited anticancer effects against pancreatic cancer in vitro through ROS-dependent apoptosis via enzyme transglutaminase 2 (TGM2)-mediated Akt/mTOR signalling. This finding is consistent with the work reported by Chen et al. [113], which found that kaempferol induces ROS production, leading to autophagy-related pyroptosis in glioblastoma cancer cells. Pyroptosis is a type of apoptosis involving proinflammatory responses and activation of the inflammasome-caspase-1-IL-1β signalling pathway [114]. Hence, it can be said that kaempferol can exert its anticancer effects through multiple mechanisms, including oxidative stress and programmed cell death pathways. Figure 8 illustrates the molecular pathway of kaempferol as an anticancer agent in various cancer cell lines.

#### 3.3.3. Fisetin

Fisetin has also been identified in Malaysian honey and possesses a diphenylpropane structure containing two aromatic rings [115]. This naturally occurring flavonoid has been proven to have extensive anticancer properties. The heterocyclic flavone backbone, which serves as the fundamental structure of this polyphenol, contains hydroxyl groups at the C-3, C-5, C-7, and C-4′ positions, all of which are essential for the compound’s biological activity [116]. According to the work by Imtiyaz et al. [117], novel propargyloxy derivatives of fisetin significantly increased cyclin B1 levels and dramatically increased the accumulation of head and neck cancer cells in the G2/M phase (*p* < 0.05). The findings demonstrated that exposure to fisetin arrests the G2/M and S phases of the cell cycle and reduces Bcl-2 protein expression. The fisetin derivative effectively induces cell apoptosis, as demonstrated by increased cyclin B1 levels. Fisetin also inhibits MTH1, which regulates apoptosis and significantly restricts the progression of skin cancer cells in the G0/G1 phase [117]. In addition, fisetin induced chromosomal abnormalities and double-strand breaks (DSBs) in TNBC cancer cells. Fascinatingly, fisetin can improve the efficacy of radiotherapy in radiosensitive TNBC by interfering with DSB repair, thereby enhancing TNBC programmed cell death [118]. By inhibiting the traditional non-homologous end-joining and homologous recombination repair mechanisms, fisetin hinders the repair of IR-induced DSB and causes chromosomal abnormality as determined by metaphase analysis [118]. In HeLa cervical cancer cells, a study by Afroze et al. [119] showed that fisetin treatment altered the protein levels of anti-apoptotic genes Bcl-2, BIRC8, MCL-1, XIAP/BIRC4, Livin/BIRC7, and clap-2/BIRC3, as well as both the transcript and protein levels of pro-apoptotic genes including APAF1, Bad, Bax, Bid, and BIK. Interestingly, these changes led to elevated levels of Caspase-3, Caspase-8, and Caspase-9, which were consistent with their expression at both the transcript and protein levels. Furthermore, fisetin suppressed the activation of the AKT and MAPK pathways, thereby inhibiting cancer cell proliferation and promoting apoptosis. By controlling the JAK-STAT/NF-kB pathways, fisetin therapy also reduces inflammation and decreases oxidative stress [119].

One drawback of fisetin is its low aqueous solubility and bioavailability, which limit its clinical effectiveness. To address this limitation, Sarvarian et al. [120] improved the drug delivery system by combining fisetin with grape-derived nanoparticles (GDN) to assess the antiproliferative and pro-apoptotic effects against MOLT-4 cells, an acute lymphoblastic leukaemia cancer cell line. It was demonstrated that fisetin-GDN reduced Bcl-2 expression and increased caspases 3, 8, and 9, as well as Bax levels, thereby inducing apoptosis in MOLT-4 cells.

#### 3.3.4. Catechin

Catechin, which is mostly found in green tea, cocoa, and other leaves or fruits, is also present in Malaysian honey, such as Gelam, Kelulut, and Acacia honey. Catechin is a flavan-3-ol, a secondary metabolite derived from polyphenols, with a structure consisting of two linked aromatic rings, a benzopyran moiety, and its derivatives [121,122]. Like other flavonoids, catechin has been reported to possess anticancer properties [123] owing to its anti-inflammatory and antioxidant effects. Several studies have explored the molecular mechanisms underlying the anticancer effects of catechin in various cancer cell lines [124,125].

Recent studies by Kuban-Jankowska et al. [126] demonstrated that catechin effectively reduces the viability of MCF-7 breast cancer cells in a dose-dependent manner. Among the catechins tested, epigallocatechin gallate (EGCG) exhibited the strongest inhibitory effect, demonstrating significant efficacy even at 15.625 µM. The anticancer mechanism includes targeting the enzymatic activity of protein tyrosine phosphatases, particularly PTP1B, a cytoplasmic phosphatase involved in cell signalling. This study showed that epigallocatechin significantly reduces PTP1B enzyme activity in breast cancer cells. Furthermore, PTP1B dephosphorylates tyrosine kinases critical for breast cancer progression, including HER1/EGFR, Src, JAK, and STAT, thereby contributing to tumour development.

The complex of (+)-catechin with two lysines (Cat:Lys) has been shown to exhibit anticancer activity against breast, colorectal, and pancreatic cancer cell lines [127]. According to Silva et al. [128], Cat: Lys reduces the uptake of ^3^H-DG, a radiolabeled glucose analogue, in MCF-7 and Caco-2 cancer cells by inhibiting non-GLUT-mediated mechanisms. The lack of effect on GLUT1 mRNA expression levels further supports this. Additionally, Cat:Lys inhibits both MCT- and non-MCT-mediated ^3^H-L uptake in MCF-7 cells while selectively inhibiting non-MCT1-mediated ^3^H-L uptake in MDA-MB-231 breast cancer cells. By simultaneously reducing ^3^H-DG and ^3^H-L uptake in MCF-7 cells, Cat: Lys potentially deprives them of glucose and lactate—two essential energy sources for cancer cell survival and proliferation. The Cat:Lys inhibited JAK2/STAT3 and the Wnt signalling pathway, thereby exerting an anti-migratory effect on breast, colorectal, and pancreatic cancer cell lines.

In other studies, Sun et al. [129] reported that catechin inhibits the proliferation of A549 non-small cell lung cancer cells in a dose-dependent manner by upregulating p21 and p27 expression while downregulating cyclin E1 expression and the phosphorylation of protein kinase (P-AKT). Meanwhile, Michel et al. [130] demonstrated that incubating catechin with the multidrug-resistant pancreatic cancer cell line EPP85-181RNOV enhances the cytotoxic effects of electrochemotherapy (ECT) using both cisplatin and calcium. The shortest incubation time (2 h) was the most effective, causing a significant decrease in cell viability at 24 h, which further declined after 48 h, indicating a loss of proliferative capacity. Their findings suggest that catechin may enhance the effects of electroporation by increasing cell permeability before the procedure, raising the electroporation threshold, and sensitising cells to chemotherapeutic agents.

#### 3.3.5. Apigenin

Another subclass of flavonoids that is well known for its anticancer properties is apigenin (4′,5,7-trihydroxyflavone). Like other flavonoids, apigenin can be extracted from edible fruits, plants, and honey, and it can be obtained as a yellow crystalline compound that is insoluble in water [131]. According to Chen et al. [132], apigenin effectively inhibits cervical cancer in vitro (HeLa and C33A cells) and in vivo (C33A xenograft tumours in mice). Furthermore, apigenin suppressed FAK signalling (involving FAK, paxillin, and integrin β1) and PI3K/AKT signalling (including PI3K, AKT, and mTOR), which modulated various downstream targets such as Bcl-2, Bax, p21cip1, CDK1, CDC25c, cyclin B1, fibronectin, N-cadherin, vimentin, laminin, and E-cadherin. This resulted in mitochondrial-mediated apoptosis, G2/M-phase cell cycle arrest, and decreased cancer cell migration, ultimately exerting anticancer effects in cervical cancer.

On the other hand, Shi et al. [133] demonstrated that apigenin’s anti-colorectal cancer (CRC) effects showed a direct correlation with pyruvate kinase M2 (PKM2) expression levels. This correlation was manifested through reduced anti-proliferative effects and diminished apoptosis induction in PKM2-knockdown LS-174T cells treated with apigenin. Lysine residue 433 (K433) was identified as the essential binding domain for PKM2-apigenin complex formation. By targeting K433, apigenin suppressed glycolytic activity in both LS-174T and HCT-8 cell lines, thereby mediating its anti-CRC effects in both experimental models.

Meanwhile, Yang et al. [54] screened multiple phytochemicals in human CRC models using HCT116 (wild-type p53) and HT29 (mutant p53) cell lines. This study showed that apigenin counteracted 5-fluorouracil (5-FU)-induced upregulation of thymidylate synthase (TS), a critical factor in chemoresistance. Apigenin also boosted ROS generation, elevated intracellular and mitochondrial Ca^2+^ levels, and increased the mitochondrial membrane potential during combination treatment with 5-FU.

Apigenin also demonstrated anticancer activity against pancreatic cancer cell lines, as reported by Feng et al. [134]. The study showed that apigenin increased NK cell proliferation by 1.7-fold and enhanced cytotoxicity against pancreatic cancer cells (IC_50_ reduction: 38%). Apigenin exerts dual immunoenhancing effects by mediating Bcl-2 upregulation and Bax suppression at both transcriptional and translational levels, and by driving perforin, granzyme B, and NKG2D expression via activation of the JNK/ERK pathway.

#### 3.3.6. Quercetin

**Figure 9:**
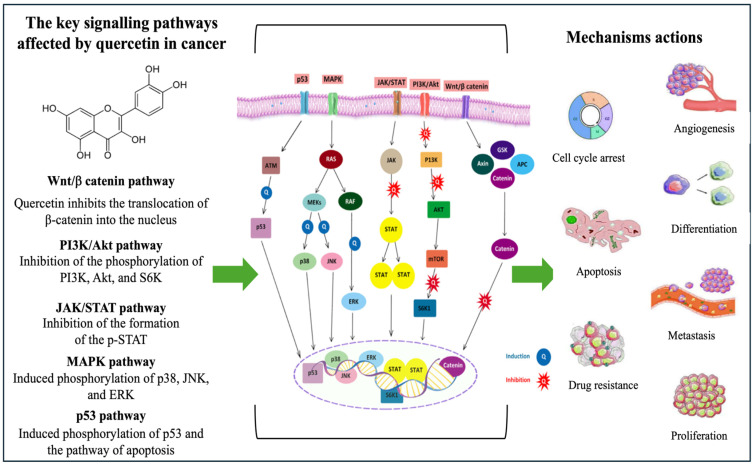
Anticancer activity of quercetin in various cancer cell lines. This figure was reproduced (adapted) from Aghababaei and Hadidi [138], an open access article published by MDPI, Basel, Switzerland, under a Creative Commons Attribution license 4.0 (CC BY 4.0).

Quercetin, scientifically known as 3,3′,4′,5,7-pentahydroxyflavone, belongs to a group of plant pigments known as flavonoids and is typically found in fruits, flowers, and vegetables [135]. Quercetin has been used in medicine for centuries due to its various health benefits. Its role in human health has been extensively studied, particularly its antioxidant, anti-inflammatory, anti-ageing, anti-microbial, anti-viral, and anti-cancer effects [136]. In anticancer applications, quercetin has been widely reported to exhibit significant anticancer activity, as shown in Figure 9. For example, the anticancer properties of quercetin have been reported to be associated with the induction of apoptosis and cell cycle arrest in various types of cancer cell lines, such as breast, colon, lung, and prostate [137].

In breast cancer treatment, targeting BRCA1 modulation could provide a better therapeutic approach for treating triple-negative breast cancer (TNBC) patients. Previous studies have demonstrated that quercetin can modulate BRCA1 expression. Studies by Kundur et al. [139] have shown that the synergistic effect of quercetin and curcumin modulates BRCA1 levels and inhibits cell survival and migration in TNBC cell lines. Additionally, studies have shown that both quercetin and curcumin significantly promote histone acetylation of the BRCA1 promoter. Furthermore, their combined treatment notably reduced cell survival and migration in ER+ cells with BRCA1 knockdown. This finding shows that the synergistic effect of quercetin and curcumin can significantly enhance anticancer activity against TNBC cells by modulating tumour suppressor genes [139].

Besides breast cancer, lung cancer is also a leading cause of cancer-related mortality and has a high global incidence rate [140]. As reported in the literature, quercetin may potentially inhibit lung cancer growth by activating the MEK–ERK pathway and inactivating Akt-1, as well as altering the expression of Bcl-2 family proteins [141]. Furthermore, quercetin may suppress the growth of various non-small cell lung cancer (NSCLC) cell lines by inhibiting overexpressed Aurora-B kinase, and thus, it holds significant promise for future lung cancer treatment [142]. Some of the limitations of quercetin include its low solubility in water (2.15 µg/mL), low bioavailability, and rapid clearance from plasma [143,144]. Therefore, modification of quercetin is required to enhance its physicochemical properties. In one study reported in the literature, Riaz et al. [145] modified quercetin using T7-targeted liposomes with different peptide densities (0.5%, 1%, and 2%), resulting in significant anticancer activity in lung cancer treatment. The in vitro studies showed that the 2% T7-QR-lip significantly augmented cytotoxicity by threefold, induced higher apoptosis, and caused S-phase cell cycle arrest. The improved anticancer performance was attributed to receptor-mediated endocytosis. These findings suggest that the modification and functionalization of quercetin with T7-targeted liposomes could serve as a promising nanocarrier for lung cancer therapy through receptor-mediated targeting at the tumour site [145].

Another study also supports the notion that quercetin, present in the traditional Chinese medicine Yang-Yin-Qing-Fei-Tang (YYQFT), has significantly contributed to the treatment of lung cancer. The active compound quercetin has been shown to significantly induce apoptosis in NSCLC, leading to a marked reduction in tumour volume without affecting the mice’s body weight. Additionally, quercetin-induced apoptosis was observed in tumour tissues. In the treatment of lung cancer, quercetin has been shown to upregulate multiple apoptosis-related genes, such as p53, Bax, and Fas, in tumour tissues, thereby increasing the Bax/Bcl-2 ratio [146].

Another type of cancer that has attracted worldwide attention is prostate cancer. According to a recent report from the National Cancer Institute’s Surveillance, Epidemiology, and End Results (NCI-SEER), prostate cancer is the most diagnosed non-cutaneous cancer among American men and is the second leading cause of cancer-related deaths in men in North America [147]. The treatment of prostate cancer involves the use of docetaxel, which was approved by the U.S. Food and Drug Administration (FDA) in 2004 and remains a standard drug in this therapy to this day. However, the limitation in using docetaxel as a therapeutic agent leads to the development of chemoresistance, thereby reducing its clinical utility [148]. It is noted that co-treatment with plant-derived bioactive compounds can help overcome chemotherapy resistance. To date, quercetin has been widely reported to have strong antioxidant activity, which could play a crucial part in chemoprevention. Furthermore, the high amount of ROS generated by quercetin could lead to oxidative stress, which may promote cell proliferation, survival, and metabolic adaptation to the tumour microenvironment by overactivating signal transduction pathways involved in tumour growth [138].

**Figure 10:**
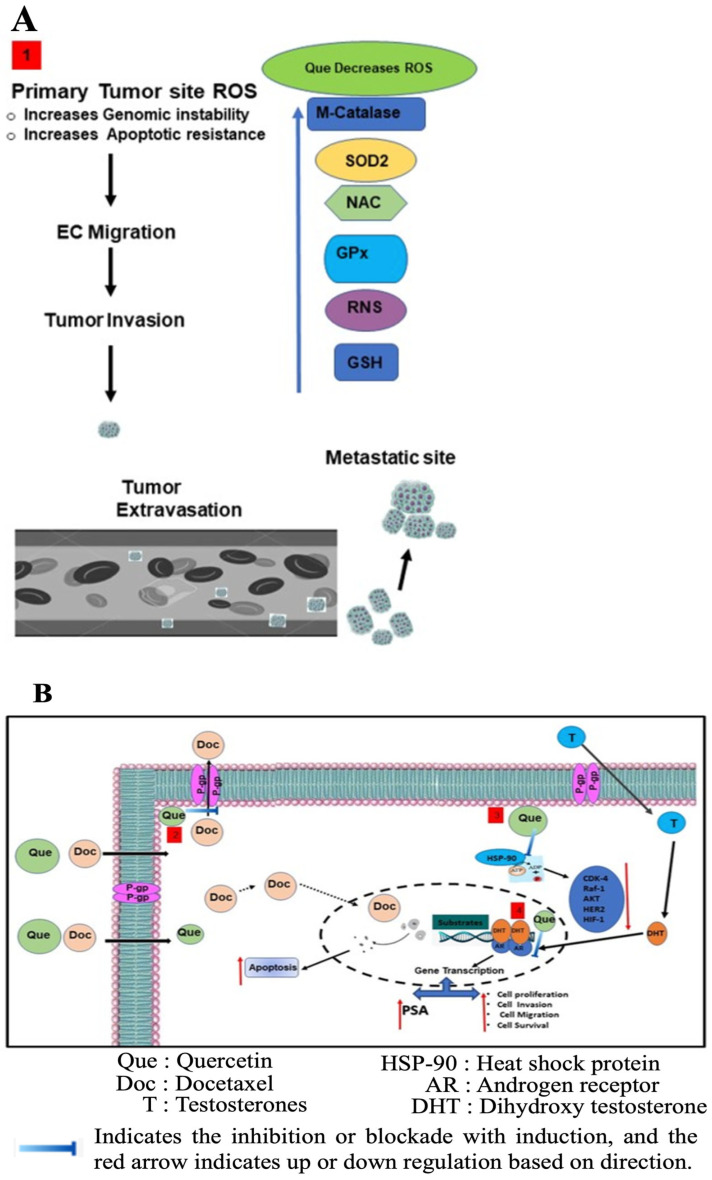
(**A**) Therapeutic effect of quercetin and (**B**) antioxidant effect of quercetin. Both figures are reproduced (adapted) from Sharma et al. [149], an open access article published by MDPI, Basel, Switzerland, under Creative Commons Attribution License 4.0 (CC BY 4.0).

Meanwhile, studies by Sharma et al. [149] showed that the most effective combination of docetaxel and quercetin was achieved when prostate cancer cell lines were pre-treated with all quercetin doses for 24 h, followed by low-dose docetaxel for another 24 h. This study showed that synergistic effects were observed at low docetaxel concentrations (0.5 and 1.0 nM) and across all tested quercetin concentrations. Figure 10A shows the potential pathways through which quercetin may synergistically amplify the effects of docetaxel, while the antioxidant properties of quercetin are shown in Figure 10B. As shown in Figure 10A, quercetin enhances ROS scavenging by upregulating enzymes involved in this process, including superoxide dismutase (SOD), glutathione peroxidase (GPx), and catalase, as well as non-enzymatically by increasing the glutathione (GSH) level. On the other hand, the antioxidant effect of quercetin (Figure 10B) reduced the levels of P-glycoprotein (P-gp), a multidrug resistance protein responsible for expelling docetaxel. The decrease led to a significant increase in the intracellular docetaxel concentration, resulting in a synergistic effect between docetaxel and quercetin. Hence, it can be concluded that quercetin significantly improves the anticancer performance of docetaxel in castration-resistant prostate cancer (CRPC). This study demonstrates a novel therapeutic approach combining docetaxel and quercetin to enhance docetaxel efficacy in CRPC treatment.

Colorectal cancer, or colon cancer, is reported to be the second leading cause of cancer-related deaths and the third most common malignancy worldwide [150]. To date, chemotherapy continues to be widely employed in the treatment of colon cancer. Although there are toxicological concerns and effects on normal cells, as well as the emergence of drug resistance, its clinical application is limited and requires safer, non-toxic approaches. In the meantime, 5-fluorouracil (5-FU) has been approved for the treatment of colon cancer. However, its toxicity to healthy tissues and the development of tumour resistance remain major challenges to effective cancer treatment. Recently, it was reported that the combination of 5-FU and quercetin improved anticancer activity compared with 5-FU alone. The synergistic effect of 5-FU and quercetin enhanced apoptosis in HCT-116 and Caco-2 cells and significantly suppressed miR-27a expression. This suppression led to the upregulation of secreted frizzled-related protein 1 and inhibition of Wnt/β-catenin signalling, as evidenced by reduced cyclin D1 expression. Hence, the presence of quercetin strongly enhances the sensitivity of 5-FU by suppressing the miR-27a/Wnt/β-catenin signalling pathway in colon cancer cells, suggesting further research on this combination at a lower 5-FU dose [151].

Other types of cancer that have shown potential for treatment with quercetin include gastric, pancreatic, and liver cancer. Gastric cancer is commonly treated with chemotherapy. However, this type of treatment has raised concerns among patients owing to its effects on normal cells and healthy tissues. Thus, a drug delivery approach employing non-ionic surfactant vesicles has been proposed to transport a range of hydrophobic and hydrophilic drugs, as well as genes, hormones, antigens, and peptides. For instance, Hemati et al. [152] successfully developed a novel cationic PEGylated niosome containing anticancer drugs (doxorubicin or quercetin) and siRNA to target both proteins and genes involved in gastric cancer development. Cell division cycle 20 (CDC20), a known oncogene, is considered a promising therapeutic target for gastric cancer. In this work, a co-delivery system for drugs and gene silencers with a high net positive charge and optimal size for siRNA loading was developed. This system, which features thermosensitive drug release, effectively silenced CDC20 expression compared with the single delivery of either siRNA or the drug alone. Moreover, the co-delivery of drugs and CDC20 siRNA exhibited a highly inhibitory effect on the growth of gastric cancer cells [152].

In pancreatic cancer therapy, quercetin has been reported to increase the sensitivity of pancreatic cancer cells to several chemotherapeutic agents, including bromodomain and extraterminal domain inhibitors (BET), doxorubicin, gemcitabine, daunorubicin, and sulforaphane. Moreover, quercetin may exert antitumor activity by preventing pancreatic cancer progression through the regulation of oxidative and inflammatory networks, thereby promoting cancer cell immune escape by inducing immunosuppressive cytokines [153]. On the other hand, the combination of quercetin with 5-FU in the treatment of liver cancer has been reported to suppress cell proliferation in a dose- and time-dependent manner. This co-treatment exerts a synergistic effect, as shown by cell cycle arrest at different phases across multiple cell lines. Hence, it can be concluded that the addition of quercetin inhibits the proliferation of liver cancer cells by inducing apoptosis and cell cycle arrest [154]. A summary of the anticancer activity of quercetin is listed in Table 2.

#### 3.3.7. Acacetin

**Figure 11:**
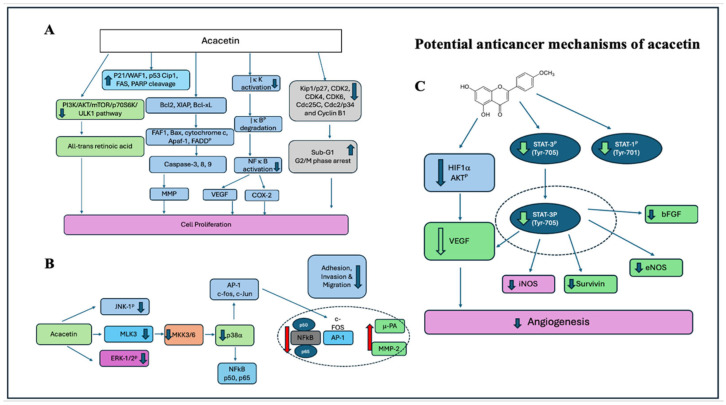
(**A**) Anticancer mechanism of acacetin. Upregulation of specific targets/transcription factors is represented by upward-pointing arrows, and downregulation by downward-pointing arrows. Outward-pointing arrows depict the chronological order of events within the mechanism. (**B**) Molecular pathway of acacetin in inhibiting the migration and invasion in cancer cells. Upward arrows indicate the upregulation of targets or transcription factors, while downward arrows indicate downregulation. Arrows pointing outward show the sequence of events in this process. (**C**) Molecular pathway of acacetin to suppress angiogenesis. Upward arrows indicate the upregulation of targets or transcription factors, while downward arrows indicate downregulation. Arrows pointing outward show the sequence of events in this process. This figure is adapted from Singh et al. [156].

Other flavonoids (C_16_H_12_O_5_), such as acacetin, have been revealed to have various biological properties, including antimicrobial, antioxidant, anti-inflammatory, and especially anti-cancer properties [155]. Therefore, reviewing its physicochemical properties and pharmacological effects is crucial for assessing its potential in cancer therapy. Acacetin has been shown to possesses high anti-proliferative potential in various cancer cells lines such as lung A549, NCI–H187 & H1299 cell, Leukaemia CLL, Jurkat T Cells, MOLT-4, HL-60, U937 and K562 cells, MOLT-4, HL-60, U937 and K562 cells, Hepatocellular carcinoma SMMC-7721, HepG2, Oesophageal squamous cell carcinoma Eca109, Prostate carcinoma PC-3M, PNT2A, DU145 and LNCaP cells, Colon Carcinoma CaCO-2, LOVO, COLO-201, HCT-8 cells, Gastric carcinoma AGS cells, Kidney HEK293T cells, and many more [156]. The proposed anticancer mechanisms of acacetin are illustrated in Figure 11.

While various reports in the literature have demonstrated acacetin’s anticancer activity across different cancer types, its specific functions and mechanisms in each cancer type remain unclear. Therefore, specific anticancer studies on major cancer cell lines were investigated. According to the literature, early studies showed that acacetin inhibits cancer cell proliferation by regulating and inducing apoptosis via caspase cascades [157] or by stimulating ROS formation [158]. Additionally, acacetin has been shown to induce cancer cell arrest in the G1 phase of liver and breast cancer cells [159], as well as in the G2/M phase of colon cancer cells [160]. Moreover, acacetin was reported to have inhibited the invasion and migration of cancer cells via regulating the PI3K/Akt/Snail pathway of gastric cancer cells [161], or by regulating the p38 MAPK signalling pathway of prostate cancer cells [162].

Recent studies have demonstrated that acacetin significantly inhibits the growth of non-small-cell lung cancer (NSCLC) cells, A549 and H460, by upregulating miR-34a, both in vitro and in vivo [163]. Further molecular studies revealed that NSCLC significantly upregulated p53 expression in both in vitro and in vivo models. On the other hand, microRNA miR-34A, which is directly regulated by p53, significantly suppresses the NSCLC cell proliferation by downregulating its target gene (programmed death ligand 1). Acacetin was also found to increase miR-34a expression and decrease programmed death ligand 1 expression in NSCLC cells. The in vitro experiment showed that silencing p53 expression with siRNAs significantly reversed the acacetin-induced increase in miR-34a and eliminated acacetin’s inhibitory effect on NSCLC cell proliferation. The studies also showed that miR-34a agomir recapitulated the effects of acacetin in A549 cells, whereas miR-34a antagomir partially or completely reversed these effects. Additionally, in vivo studies revealed that intratumoral injection of a miR-34a antagomir significantly attenuated the anti-tumour effects of acacetin in A549 Xenograft nude mice. These findings suggest that acacetin inhibits NSCLC cell proliferation and induces apoptosis by regulating miR-34a [163].

Previous studies reported that acacetin could induce cancer cell arrest in the G1 phase of liver and breast cancer cells [159]. However, the pathway by which acacetin exerts its anticancer effects on breast cancer cells remains unclear. Recently, Kandhari et al. [164] explored the mechanisms of cell death and growth inhibition induced by acacetin in breast carcinoma T-47D and MDA-MB-231 cells. The study demonstrated that acacetin, at concentrations ranging from 10 to 40 μM, decreased the levels of G2/M-phase cyclins and CDKs while upregulating CDK inhibitors, such as Cip1/p21. In addition, a concentration-dependent increase in cell death was observed in both breast cancer cells, with minimal effects on the non-tumorigenic MCF-10A breast cells. Furthermore, acacetin induced mitochondrial superoxide production, DNA damage, and ROS. Pre-treatment with N-acetyl cysteine (NAC) suppressed ROS generation and partially reversed ERK1/2 activation. Furthermore, acacetin has been shown to upregulate RIP1 and RIP3 expression. Treatment with the RIP1-specific inhibitor Necrostatin-1 (NS-1) effectively counteracted acacetin-induced DNA damage and cell death. Hence, these results suggest that acacetin exerts its anticancer effects by inducing ROS and RIP1-dependent necroptosis in breast cancer cells.

Previous studies by Kandhari et al. [165] using acacetin against breast cancer cell lines MCF-7 and MDA-MB-468 also showed positive results. The viability of the cancer cells, evaluated by the MTT assay, decreased significantly from 41% to 76%. The significant anticancer activity of acacetin was due to its strong growth-inhibitory effect, as confirmed by the trypan blue assay. Additionally, acacetin has been shown to inhibit the migratory potential of breast cancer cells in a wound-healing assay, while Western blotting revealed a downregulation of vimentin and N-cadherin. Furthermore, flow cytometric analysis revealed cell cycle arrest, with acacetin significantly inhibiting ERK1/2 and AKT signalling and modulating the expression of cell cycle regulators. This study showed that acacetin significantly reduces cancer cell survival, leading to cell cycle arrest and inhibiting both migratory potential and EMT in breast cancer cells.

Acacetin also demonstrated potential as an anticancer agent against colon cancer cells. For example, Prasad et al. [166] showed its efficacy against two colorectal adenocarcinoma cell lines, SW480 and HCT-116. The studies also showed that acacetin significantly decreased cell survival and proliferation in both cell lines. while inducing S- and G2/M-phase arrest. Moreover, acacetin has been shown to lower β-catenin and c-My levels. Analysis using Annexin V-FITC and nuclear condensation revealed that acacetin induced apoptosis and increased mitochondrial ROS production. Additionally, acacetin caused significant MMP depolarisation and increased the Bax/Bcl-2 ratio. In this study, no changes were observed in caspases-8 and caspases -9 or PARP levels. However, acacetin was found to induce the truncation and subsequent translocation of activated AIF from the mitochondria to the cytosol, which would further lead to chromosomal breakage and apoptosis [166].

Likewise, acacetin exhibits anticancer activity by targeting multiple pathways. According to Yun et al. [167], acacetin acts as a STAT3 inhibitor and binds directly to STAT3 using various biochemical approaches. Moreover, acacetin inhibited STAT3 phosphorylation at tyrosine 705 and prevented its nuclear translocation in DU145 cells. This led to the downregulation of STAT3 target genes and induced apoptosis in a time-dependent manner. The study further revealed that although acacetin promoted ROS generation, this effect was not implicated in STAT3 inhibition, as suppression of p-STAT3 levels was not reversed by treatment with ROS inhibitors such as GSH or NAC. Furthermore, acacetin was reported to inhibit tumour growth in nude mice xenografts. Hence, this suggests that acacetin, as a STAT3 inhibitor, holds great promise as a potential drug candidate for targeting STAT3 in cancer therapy [167].

Additionally, the effects of acacetin on STAT3 activation, protein kinases, phosphatases, products of STAT3-regulated genes, and apoptosis were also investigated in human hepatocellular carcinoma cell lines [168]. In hepatocellular carcinoma cells, acacetin suppressed STAT3 activation in a dose- and time-dependent manner, and also inhibited upstream kinases, including c-Src, JAK1, and JAK2. Moreover, vanadate treatment reversed the STAT3 inhibition induced by acacetin, suggesting a role for tyrosine phosphatase activity in this process. By inhibiting STAT3, acacetin also significantly reduced the expression of proteins involved in proliferation, survival, and angiogenesis. Hence, this finding strongly supports the potential of acacetin as a novel STAT3 inhibitor [168].

Epithelial–mesenchymal transition (EMT) is a primary cause of recurrence and metastasis in postoperative gastric cancer patients. Few studies have demonstrated that acacetin significantly suppresses cell adhesion and focal adhesion formation. Thus, exploring how acacetin influences EMT is crucial for elucidating its anticancer activity in gastric cancer. For example, Zhang et al. [161] studied the role of acacetin in EMT in human gastric cancer cell lines (MKN45 and MGC803) through both in vivo and in vitro experiments. The findings revealed that acacetin significantly inhibited the proliferation, invasion, and migration of human gastric cancer cells by regulating the expression of EMT-related proteins. On the other hand, acacetin was observed to reverse the morphological changes from epithelial to mesenchymal cells in the TGF-β1-induced EMT models. Additionally, it significantly suppressed PI3K/Akt signalling activation and reduced phosphorylation levels in TGF-β1-treated cancer cells. Meanwhile, studies also revealed that acacetin slowed the progression of peritoneal metastasis in gastric cancer models using nude mice. Furthermore, restricting the expression of EMT-related proteins limited liver metastasis. Hence, it can be concluded that acacetin significantly suppresses invasion, metastasis, and TGF-β1-induced EMT in gastric cancer, most likely by inhibiting the PI3K/Akt/Snail signalling pathway.

Ovarian cancer is another type of cancer characterised by high metastatic potential and a recurrence rate of approximately 50–85% within 5 years, affecting a significant proportion of patients [169]. Studies by Tian et al. [170] demonstrated that acacetin inhibited cell proliferation and invasion in ovarian cancer cells while promoting apoptosis. Furthermore, acacetin downregulated mesothelial cell-induced transcripts and reduced the production of pro-inflammatory cytokines IL-6 and IL-8. It also inhibited lysophosphatidic acid (LPA) production in mesothelial cells, thereby blocking the activation of the RAGE-PI3K/AKT signalling pathway in ovarian cancer cells. Table 3 summarises the anticancer properties of quercetin.

#### 3.3.8. Pinocembrin

**Figure 12:**
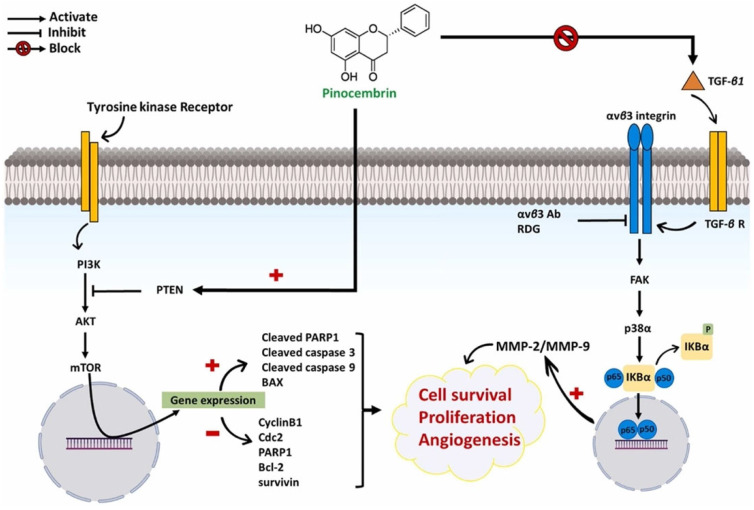
Anticancer mechanisms of pinocembrin. Pinocembrin suppresses cancer development by inhibiting the growth and spread of cancer cells via upregulation of PTEN, thereby inhibiting the PI3K/AKT/mTOR pathway. Additionally, it prevents TGF-β1-triggered cancer progression by interacting with αvβ3 integrin, thereby activating FAK and p38α. This activation enables p50 and p65 to move into the nucleus, increasing MMP-2 and MMP-9 levels that support cancer growth and metastasis. This figure is reproduced from Elbatreek et al. [176], an open access article published by Elsevier BV, under Creative Commons Attribution license 4.0 (CC BY 4.0).

Pinocembrin (C_15_H_12_O_4_) is a class of flavonoid that is widely distributed in various plants, fungi, and bee-derived products such as honey. Similar to other flavonoids, it exhibits a broad spectrum of pharmacological activities, including antioxidant, anti-inflammatory, antimicrobial, and anticancer effects [171]. This section provides a comprehensive review of pinocembrin’s anticancer properties and its mechanisms of action against multiple cancer cell lines. Over the past few years, pinocembrin has been highlighted in several recent studies for its potential therapeutic efficacy in cancer treatment. Various preclinical investigations on pinocembrin have provided insights into its activity in cancer therapy [172]. For example, Kumar et al. [173] showed that pinocembrin exerted cytotoxicity by stimulating the proapoptotic Bax protein in colon cancer cells (HCT116). This resulted in the activation of caspases 9 and 3 and the disruption of MMP (Figure 12). Meanwhile, pinocembrin was also reported to significantly suppress TGF-β1 in human retinoblastoma cells, thereby inhibiting the αvβ3 integrin/FAK/p38α signalling pathway. This inhibition reduced epithelial–mesenchymal transition and cell metastasis [174]. In melanoma cells, the flavonoid exhibited dose-dependent apoptotic activity by inducing endoplasmic reticulum stress through the inositol-requiring transmembrane kinase/endoribonuclease 1α/X-box binding protein 1 (IRE1α/Xbp1) pathway. Additionally, it suppressed autophagy by activating the PI3K/Akt/mTOR pathway (Figure 12) [175]. Overall, these data provide insight into how pinocembrin functions as an anticancer agent, leading to further, more detailed studies in specific cancer cell lines.

Recently, the anticancer properties of pinocembrin have been studied across multiple cancer cell types. For example, studies by Zhu et al. [177] demonstrated that pinocembrin exhibited significant antiproliferative and antimetastatic effects in vitro, as well as inhibited tumorigenesis in a mouse model of breast cancer (MCF-10A cells, breast cancer cell lines MCF-7, SKBR3, and MDA-MB-231). Additionally, pinocembrin may suppress breast cancer development by modulating the PI3K/AKT pathway. Its antiproliferative effects in breast cancer cells are attributed to G2/M phase cell cycle arrest and the promotion of apoptosis. Treatment with pinocembrin downregulated proteins associated with cell cycle progression and apoptosis, including cyclin B1, Cdc2, PARP1, Bcl-2, and survivin, while upregulating cleaved PARP1, cleaved caspase-3, cleaved caspase-9, and BAX. Moreover, pinocembrin effectively inhibited the growth of MCF-7 cells using a murine subcutaneous tumour model. Pinocembrin also significantly inhibited breast cancer cell migration and invasion when tested at low concentrations, suggesting that this effect involves inhibition of the PI3K/AKT pathway.

**Figure 13:**
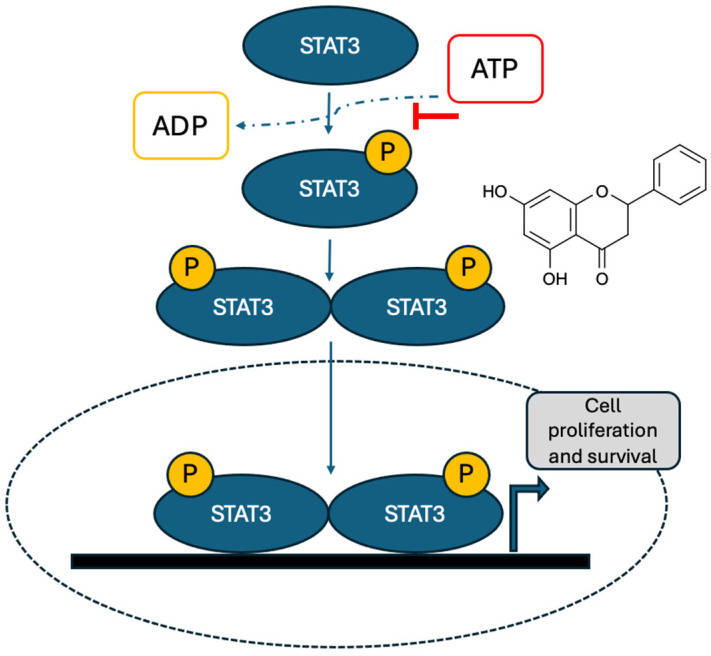
Pinocembrin inhibits STAT3 phosphorylation, leading to downregulation of downstream target genes, such as cyclin D1, which, in turn, suppresses cyclin E expression. STAT3 inhibition reduces cell proliferation and survival. The figure is adapted from Saengboonmee et al. [178].

Few studies in the literature have reported the ability of pinocembrin to significantly suppress liver cancer, specifically hepatocellular carcinoma (HCC). For example, Saengboonmee et al. [178] demonstrated that pinocembrin exhibited inhibitory effects on the proliferation of two HCC cell lines, HepG2 and Li-7. Flow cytometry analysis revealed that pinocembrin induced G1-phase cell cycle arrest in hepatocellular carcinoma (HCC) cells, with only a modest effect on cell death. Pinocembrin was also shown to inhibit STAT3 by binding to it with higher affinity than Stattic, a known STAT3 inhibitor, and by suppressing STAT3 phosphorylation at both Tyr705 and Ser727 residues. Consequently, the expression of STAT3-regulated cell cycle proteins, including cyclin D1, cyclin E, CDK4, and CDK6, was significantly reduced. The pathway summary is illustrated in Figure 13.

Besides liver cancer, pinocembrin has also been reported to have a significant impact on colorectal cancer. According to Jiang et al. [179], pinocembrin inhibited the viability, migration, invasiveness, and expressions of MMP-2 and N-cadherin in colorectal cells, while simultaneously promoting E-cadherin and lactamase beta (LACTB) expressions. Additionally, silencing LACTB increased viability, migration, and invasiveness, and elevated MMP-2 and N-cadherin levels in CRC cells, while reducing E-cadherin expression. Pinocembrin reversed these changes; however, its anticancer effects on CRC cells were partially attenuated by LACTB silencing.

Pinocembrin also shows potential for treating ovarian cancer; however, its specific role in this context requires further elucidation through both in vivo and in vitro experiments. The specific role of pinocembrin in cancer treatment can be investigated by integrating network pharmacology and molecular docking. To achieve this, Wang et al. [180] compiled pinocembrin targets from various online databases and identified ovarian cancer targets using the GeneCards database, determining common target genes through data aggregation. Protein–protein interactions were examined using the STRING platform, followed by analyses of Gene Ontology and Kyoto Encyclopaedia of Genes and Genomes (KEGG) pathways. Molecular docking was performed to assess the binding affinity between active compounds and potential targets. Meanwhile, the accuracy of these targets was validated by using in vitro. A total of 163 potential pinocembrin targets for the treatment of ovarian cancer were identified. The results indicated that pinocembrin is involved in protein kinase activity, protein phosphorylation, and cancer-related pathways. The strong binding affinities of pinocembrin with the target sites were confirmed via molecular docking. Furthermore, in vitro studies demonstrated that pinocembrin induces apoptosis in ovarian cancer cells through modulation of the AKT1–mTOR signalling pathway [180].

In another study, pinocembrin also exhibited high anticancer activity against the PC-3 prostate cancer cell line. Zhu et al. [177] found that pinocembrin inhibited cell proliferation and reduced cancer colony formation in PC-3 cells in a dose-dependent manner by modulating the expression of caspase-3, caspase-9, Bax, and Bcl-2, thereby inducing apoptosis in PC-3 prostate cancer cells. Meanwhile, pinocembrin also induced G0/G1 phase cell cycle arrest in a dose-dependent manner. Hence, it can be concluded that pinocembrin exhibits strong anticancer activity in prostate cancer by inducing apoptosis, promoting ROS production, and causing G0/G1 phase cell cycle arrest. In another work, Zhang et al. [181] found that pinocembrin can regulate the STAT3 pathway, inhibiting the migration and invasion of non-small cell lung cancer (NSCLC) cells (A549). At 50 μM, pinocembrin significantly suppressed cell migration and invasion by upregulating E-cadherin while downregulating N-cadherin and vimentin. It also blocked STAT3 phosphorylation and activation. However, overexpressing STAT3 reversed pinocembrin’s inhibitory effects on cell migration and invasion.

In summary, pinocembrin exhibits significant anticancer activity by suppressing cancer cell proliferation, migration, and metastasis, and promoting apoptosis through mechanisms that target cell cycle and signalling pathways. Further experimental investigations are necessary before proceeding to clinical trials. Table 4 summarises the anticancer properties and mechanisms of action of pinocembrin against selected cancer types.

#### 3.3.9. Pinobanksin

Pinobanksin, also known as (2R,3R)-3,5,7-Trihydroxyflavan-4-one, is a flavonoid found in natural sources. It has been shown to possess a range of pharmacological activities [183], including antioxidant effects [184]. Pinobanksin demonstrates potential as a compound anticancer agent by inducing apoptosis, inhibiting cell proliferation, and modulating cancer-related signalling pathways, making it a potentially valuable component for cancer treatment or prevention [185]. However, compared to other flavonoids, there is limited information about pinobanksin’s anticancer properties in the literature. Only a few studies have reported the potential of pinocembrin as an anticancer agent, while most research focuses on propolis, a resinous substance collected by bees that contains high levels of flavonoid compounds, including pinocembrin and chrysin.

Studies by Chen et al. [186] demonstrated that pinobanksin effectively inhibits the proliferation of SH-SY5Y cells, a neuroblastoma cell line derived from a metastatic bone tumour, by interacting with BAX, BCL-2, and CDK4/6. This interaction enhances hydrogen bonding, thereby inhibiting the activities of these proteins. Meanwhile, studies by Melekoğlu et al. [187] showed that pinobanksin at low doses has a significant proliferative effect on breast cancer cell lines (MCF-10A). On the other hand, pinobanksin has also been reported to have antiangiogenic potential in both in vitro and in vivo assays, a characteristic that could slow or stop tumour growth by cutting off its blood supply. According to Bang and Ahn [183], pinobanksin inhibits tube formation and induces apoptosis with partial fragmentation of endothelial cells. Moreover, Western blot analysis showed that pinobanksin inhibits angiogenesis by inactivating the Raf/MEK/ERK pathway and activating apoptotic signals, including caspase cascades. Hence, suggesting the ability of pinobanksin to exert antiangiogenic effects by triggering apoptosis in endothelial cells, and has significant potential to be developed as an antiangiogenic agent.

Propolis, a resin material collected by bees that contains flavonoids such as pinobanksin, has been widely investigated for its pharmacological activities. Recent studies by Moskwa et al. [188] demonstrated that propolis exhibits significant anticancer properties, showing anti-proliferative effects on astrocytoma cells (DASC, T98G, and LN-18) through apoptosis induction, cell cycle arrest, and attenuated migration. These findings suggest that propolis, rich in compounds such as pinocembrin, pinobanksin, pinobanksin 3-acetate, and chrysin, may be a promising anticancer candidate for glioblastoma therapy. Other studies, such as those by Tumbarski et al. [189], have investigated the potential of propolis as an anticancer agent against breast cancer cells (MDA-MB-231). These studies reported IC50 values ranging from 9.24 to 13.62 µg/mL as evaluated by the MTT assay, indicating enhanced anticancer activity. The improved anticancer activity is associated with its capacity to inhibit cell proliferation, suppress tumour growth, and reduce cancer cell invasion across multiple cancers. Similar findings were also obtained when propolis was used as an anticancer agent against various cancer cell lines, including breast adenocarcinoma (MCF7), cervical carcinoma (HeLa), hepatocellular carcinoma (HepG2), malignant melanoma (MM127), and NSCLC (NCI-H460) [190]. Table 5 summarises the anticancer properties and pathway of pinobanksin and propolis.

### 3.4. Other Flavonoid Compounds

#### 3.4.1. Hesperetin

Hesperetin (C_16_H_14_O_6_) is a naturally occurring flavone and polyphenolic compound present in citrus fruits such as lemons and oranges. Recently, hesperetin has attracted significant interest among researchers due to its anti-tumour characteristics. Hesperetin demonstrates a cytotoxic action against various types of cancer cells, including breast cancer, prostate cancer, glioblastoma, liver cancer and many more [191]. The primary benefit of utilising hesperetin cancer treatment lies in its natural availability, structural chemistry, ability to generate nano-carriers to enhance absorption, pharmacokinetic profile, and reduced toxicity [192]. Furthermore, research towards chemotherapy agents shows hesperetin might have the potential to be developed as a co-chemotherapeutic agent combination with doxorubicin, resulting in arresting the cell cycle, inducing apoptosis, suppressing Rac1, HER2, MMP9 expression, and cell migration in HER2 overexpressing breast cancer cells respective doses of 95 μM and 0.2 μM, where dose were 377 and 0.8 μM, respectively during individual testing [193]. Although hesperetin offers numerous therapeutic benefits, it may also address certain undesirable effects or toxic reactions. Rajasekar et al. [194] reported that hesperetin treatment causes oedema in the yolk sac and pericardial cavity, decreased heartbeat rate, upcurved tail, cardia chamber bulging, and curved body axis in the Zebrafish model. In terms of its capability in alleviating multidrug resistance in numerous cancer types, study has shown hesperetin combined with 5-fluorouracil (5-FU) was more effective rather than a single use of the drug in Eca-109 cell line and a xenograft mouse model of oesophageal cancer through down-regulating Bcl-2 with simultaneously increasing Bax, caspase-3, caspase-9 more effectively than did 5-FU only and reduced cancer cell invasion with cell proliferation through mitigating PI3K/AKT signalling pathway [193].

#### 3.4.2. Naringenin

Similar to hesperetin, naringenin (C_15_H_12_O_5_) is a flavonoid found in grapefruit and other citrus fruits that exhibits antioxidant and anticancer properties. Its anticancer activity is largely attributed to its ability to inhibit cell proliferation and induce apoptosis. In general, the anticancer properties of naringenin encompass oxidative stress and inflammation by reducing pro-inflammatory cytokines and improving antioxidant parameters, thereby disrupting cancer development [195]. Few studies have reported naringenin’s ability to interfere with cancer growth. For instance, naringenin has been reported to successfully suppress the development of gastric carcinoma induced by N-methyl-N′-nitro-N-nitrosoguanidine, partly due to its antioxidant properties. Moreover, naringenin suppresses the formation of MDA-DNA complexes generated in BaP-induced lung carcinogenesis [196]. It has been reported that naringenin-induced oxidative stress causes toxic effects in various cancer cell types. For instance, in placental choriocarcinoma, naringenin induces oxidative stress and subsequent apoptosis via ROS-dependent activation of ERK 1/2 [197]. Moreover, naringenin has been shown to act as an anticancer agent by inducing oxidative stress. This is attributed to its inhibitory actions on the antioxidant enzyme glutathione reductase [198]. It is important to note that naringenin shows great promise as an adjuvant treatment for cancer and can enhance the effectiveness of other chemotherapy drugs when combined. In fact, naringenin, by elevating ROS levels, potentiates tamoxifen’s action in breast cancer cells [198], as well as enhances paclitaxel inhibition of prostate cancer progression [199].

#### 3.4.3. Vitexin

Vitexin (Apigenin-8-C-β-D-glucopyranoside) is a C-glycosylated flavone featuring apigenin with glucose bound via a stable C-C linkage at position 8 and can be found in passionflower, hawthorn, mung bean, and bamboo leaves. Studies have shown that it exhibits potent antioxidant, anti-inflammatory, neuroprotective, anticancer, cardioprotective, and metabolic regulatory activities [200]. According to the literature, vitexin has been demonstrated to reduce the viability of HEC-1B (IC_50_ = 9.89 μM) and Ishikawa (IC_50_ = 12.35 μM) endometrial cancer cells. Furthermore, the PI3K/Akt activator 740Y-P (20 μM) reversed the inhibitory effects of vitexin on these cells. Meanwhile, a 30-day xenograft tumour study showed that vitexin (80 mg/kg) suppressed tumour growth in an endometrial cancer model. Immunohistochemistry analysis indicated that vitexin reduced the expression of Ki-67, OCT4, and VEGFA in tumour tissues and significantly inhibited the phosphorylation of PI3K and AKT following the complete treatment. Thus, these findings demonstrate that vitexin effectively suppresses tumour growth in vivo in endometrial cancer studies [201].

#### 3.4.4. Isoorientin

Isoorientin (C_21_H_20_O_11_), a C-glucosyl flavone antioxidant, features luteolin with a glucose attached via a stable C-C bond at position 6. It can be found in buckwheat, olive leaves, and herbs and demonstrates anti-inflammatory, neuroprotective, and anticancer activities by inhibiting cancer cell growth, reducing diabetes-related complications, and protecting against liver damage [202]. Isoorientin induced cell apoptosis through ROS-mediated MAPK/STAT3/NF-κB signalling pathways and inhibited cell migration by regulating the AKT/GSK-3β/β-catenin signalling pathway in gastric cancer AGS cells [203]. Activation of the MAPK signalling pathway plays a key role in the induction of cancer cell apoptosis by natural compounds [204]. Recent evidence indicates that the MAPK, STAT3, and NF-κB signalling pathways are critical regulators of cell apoptosis [205]. This finding demonstrated that isoorientin can activate the p38 and JNK pathways while suppressing the ERK, STAT3, and NF-κB signalling pathways. Moreover, another study reported that isoorientin upregulated intracellular ROS levels, induced G2/M cell cycle arrest, and triggered apoptosis by regulating the MAPK/STAT3/NF-κB signalling pathway in A549 human lung cancer cells, suggesting its potential as a therapeutic agent for lung cancer [206]. Furthermore, previous studies have demonstrated that isoorientin inhibits colorectal cancer cell proliferation by regulating the cell cycle and modulating the expression of genes involved in apoptosis. These findings suggest that ISO may also serve as a therapeutic agent for CRC treatment [207].

#### 3.4.5. Xanthohumol

Xanthohumol, a prenylated chalcone from *Humulus lupulus* female inflorescences, features two aromatic rings linked by an α,β-unsaturated ketone, a methoxy group, multiple phenolic OH groups, and a 3,3-dimethylallyl (prenyl) side chain. This structure confers potent anticancer, anti-inflammatory, and antioxidant activities [208]. Recent studies have shown that xanthohumol inhibits growth and induces apoptosis and caspase-dependent cell death in HepG2 cells via the NF-κB/p53-apoptosis signalling pathway. It also increases the activity of the Bax/Bcl-2-cytochrome c-caspase-3-PARP and AIF pathways while suppressing the XIAP signalling pathway. The findings confirm the anticancer effect of xanthohumol in human liver cancer [209]. Previous studies showed that xanthohumol induced cell death and morphological changes in Ca Ski cells, as observed by phase-contrast microscopy and fluorescent staining [210]. Furthermore, the recent study highlights the anti-cancer activities of xanthohumol, a natural bioactive derived from the Humulus lupulus, against breast and lung cancer cells in 3D cell culture [211]. The findings revealed that xanthohumol effectively induces apoptosis, halts cell cycle progression, suppresses invasion, and mitigates drug resistance in breast and lung cancer cells. Many studies have reported that various natural compounds and polyherbal formulations can inhibit drug resistance and radioresistance in cancer cells [212,213]. Thus, xanthohumol may exert anticancer effects in colon cancer by enhancing the DNA damage response and apoptosis, as well as activating the ataxia telangiectasia mutated (ATM) pathway. The ability of xanthohumol to promote DNA repair resulted in the chemosensitisation of CRC cells to the chemotherapeutic agent SN38 (7-ethyl-10-hydroxycamptothecin) [214].

#### 3.4.6. Galangin

Galangin, an active flavonoid isolated from the root of the *Alpinia officinarum Hance*, has a cytotoxic effect on many cancer cell lines in vitro [215]. Numerous studies have revealed that galangin has a comprehensive range of anti-tumour effects, including colon cancer, breast cancer, leukaemia, lung cancer, oesophageal cancer and hepatocellular carcinoma (HCC) [216,217]. A recent study using galangin as a treatment for HCC reports that it inhibits multiple signalling pathways, including the PI3K-Akt pathway, cell cycle, and cancer pathways, as indicated by GO and KEGG enrichment analyses [218]. A recent study indicated that the miR-21-mediated PTEN/AKT pathway plays an important role in the anti-tumour effects of galangin on cholangiocarcinoma (CCA) cells [205].

## 4. Therapeutic Potential for Malaysian Honey as an Anticancer Agent

Malaysian honey, particularly Tualang, Gelam, Kelulut, and Acacia, shows promise as a therapeutic candidate for anticancer treatment through various mechanisms and molecular pathways, as discussed in this review. Given the rich phytochemical content, especially polyphenols and flavonoids, in various types of Malaysian honey, it holds promising potential as an anticancer agent. For example, honey is said to have high antioxidant activity and anti-inflammatory effects, reducing oxidative stress and modulating tumour-promoting inflammation, both of which are considered crucial for cancer prevention and therapy.

Moreover, the phenolic and flavonoid compounds present in Malaysian honey can induce apoptosis in various cancer types through mechanisms such as mitochondrial membrane depolarisation, caspase activation, and regulation of apoptotic proteins, thereby promoting programmed cancer cell death while sparing normal cells. This has been reported in the literature where Tualang honey, which is rich in phenolic and flavonoid compounds, significantly induces apoptosis in human breast and cervical cancer cells via mitochondrial membrane depolarisation and activation of caspase pathways [15,219]. In addition to Tualang honey, other Malaysian honeys, such as Gelam and Nenas honey, were also successfully reported to be capable of inhibiting the growth of colon cancer cells by triggering programmed cell death and reducing inflammation [220].

Furthermore, the presence of these bioactive compounds significantly suppresses cancer cell growth by promoting cell cycle arrest and reducing tumour growth in various types of cancer, including breast, cervical, leukaemia, oral, lung, and pancreatic cancers, both in vitro and in animal models. It also reduces angiogenesis and may inhibit metastasis, limiting cancer spread. Several studies have also shown that Malaysian honey can enhance the effectiveness of chemotherapeutic agents and reduce side effects, thanks to its bioactive components. One unique property of Malaysian honey is its low or non-toxicity to normal cells, suggesting its safety potential. Hence, Malaysian honey has promising potential as a multifunctional anticancer agent with demonstrated efficacy in vitro and in animal models. However, further experimental and clinical studies are required to confirm its pathways and mechanisms, optimise formulations and dosages, and validate its use as an adjuvant or alternative in cancer therapy.

## 5. Challenges and Limitations of Honey as an Anticancer Agent

Although honey has demonstrated numerous health benefits and medicinal properties, its classification as a food supplement has also been associated with certain infections and adverse health effects [221]. Thus, several challenges and limitations need to be addressed before honey-based therapies can be established as an alternative cancer treatment. For example, honey consumption has been linked to allergic reactions [222], abdominal discomfort [223], and the risk of infant botulism [224]. Moreover, the concentration and variability of phytochemicals present in honey [21] may significantly influence its consistency and effectiveness in cancer treatment. Therefore, it is highly recommended to standardise honey extracts to ensure consistent therapeutic outcomes [225]. In addition, the precise molecular pathways of honey are not yet fully understood; thus, further studies are required to confirm its mechanisms and interactions. Furthermore, there is currently a lack of robust evidence from rigorous prospective, randomised clinical trials confirming the effectiveness of honey in clinical oncology, highlighting an important opportunity for future investigation. Nevertheless, this review provides fundamental insights into how individual phytochemical compounds in honey modulate key molecular signalling pathways involved in cancer. By elucidating these mechanisms, this work establishes a foundation for the potential development of specific honey-derived compounds as novel anticancer agents.

Another major challenge is the bioavailability and bioaccessibility of honey, particularly its polyphenols. Factors such as floral origin, processing, and digestion conditions can significantly influence the amount absorbed by the body [226]. Studies indicate that phenolic compounds often exhibit high bioaccessibility due to structural transformations during digestion [227]. However, other components, such as α-dicarbonyls, demonstrate low bioaccessibility [226]. To overcome these limitations, drug delivery systems using nanoparticles can be recommended [228]. However, the preparation and optimisation of such systems require a rigorous and systematic evaluation process to ensure both safety and regulatory compliance [229].

Furthermore, the incorporation of honey as an anticancer agent into clinical settings faces several regulatory and acceptance barriers [230]. While there is evidence supporting the application of honey in the treatment of wounds and up to second-degree burns [231], there remains a lack of evidence demonstrating its successful use in clinical oncology. Consequently, this has led to regulatory challenges that may limit its clinical applications [230]. In addition, obtaining approval and community acceptance for the use of honey as an anticancer agent requires a shift in perspective, supported by robust scientific evidence and a clear clinical practice framework.

## 6. Conclusions

Malaysian honey, enriched with various phytochemical compounds, shows promising potential as an anticancer agent to address the limitations of conventional therapies. Although the complete mechanisms remain unclear, these compounds have demonstrated activity through multiple molecular pathways in cancer treatment, including the induction of programmed cell death, suppression of tumour growth, and enhancement of chemotherapeutic efficacy, while exhibiting minimal toxicity to normal cells. In summary, by modulating various targets, honey may inhibit cancer growth through interactions with BAX, Bcl-2, and CDK4/6; suppression of the PI3K/Akt/Snail signalling pathway; inhibition of ERK1/2 and AKT signalling; silencing of CDC20 expression; suppression of the miR-27a/Wnt/β-catenin pathway in CRC; activation of the MEK–ERK pathway; inactivation of Akt-1; and alterations in Bcl-2 family protein expression. All of these findings highlight its potential as a natural and multifunctional anticancer candidate.

Despite the promising evidence from preclinical, in vivo, and in vitro studies, further experimental and clinical research is urgently required to fully elucidate its molecular pathways, mechanisms, drug delivery efficiency, toxicity, and biocompatibility, in order to ensure safe and practical applications that could complement or potentially replace current conventional cancer therapies. Other issues, such as the varying anticancer activities of honey from different floral sources and the question of why honey exhibits anticancer properties despite containing sugar, which is generally considered carcinogenic, require further investigation. Moreover, findings from in vivo and in vitro studies or animal models cannot be directly applied to humans. Clinical trial designs, including prospective, randomised trials, are required to confirm the efficacy of honey as an alternative cancer treatment.

## Figures and Tables

**Table 1:** Summary of the anticancer properties of Syringic Acid.

Cancer Type	Findings	References
Colorectal cancer cells (SW-480)	Modulated the expression of proliferative genes, including p53, ERK1/2, AKT, PI3K, and NF-κB, in SW-480 cells. Additionally, syringic acid activated autophagy in SW-480 cells.	[78]
Gastric cancer cell lines (AGS)	Inducing apoptosis, inhibiting inflammation, and modulating the mTOR/AKT signalling pathway.	[80]
Lung cancer cell (A549)	Induced moderate ROS generation in a concentration-dependent manner, disrupted mitochondrial membrane potential, and caused morphological modifications.	[81]
Liver cancer (HepG2 Cells)	Syringic acid treatment significantly increased the expression of apoptotic markers, including caspases 3 and 9, cytochrome c, Apaf-1, Bax, and p53, in HepG2 cells. This upregulation suggests that syringic acid may induce apoptosis in these cells.	[82]
Breast cancer cell (In silico studies)	Syringic acid achieved the highest docking scores with PR and HER-2 proteins, at −7.7 kcal/mol	[83]
In silico studies	Syringic acid and its derivative SA10 bind to the active site of NF-κB, thereby interfering with the association between DNA and NF-κB. SA10 exhibits a more robust binding affinity than SA and is firmly docked into the interior of NF-κB,	[84]
Leukemia (K562 Cells)	SA10 exhibited a two-fold increase in inhibitory activity compared to SA. SA10 functions as both an NF-κB inhibitor and an apoptosis inducer, a significant finding given NF-κB’s role in promoting cancer cell survival and proliferation.	[84]
Kidney and liver cancer cells (HEK 293 and HepG2 cells)	Addition of Cu (II) to Syringic Acid significantly reduced HepG2 cell viability after 72 h, increasing ROS formation, apoptosis, and autophagy without affecting mitochondrial integrity. This combination showed pro-oxidant activity, reduced cell survival, and induced autophagy in cancer cells with minimal effects on normal cells, making it a promising therapeutic candidate for cancer treatment.	[87]

**Table 2:** Summary of anticancer activity of quercetin.

Cancer Type	Findings	References
Breast Cancer	Quercetin and curcumin induce histone acetylation at the BRCA1 promoter, reducing cell survival and migration in ER+ cells by counteracting the effects of BRCA1 knockdown.	[139]
Lung cancer	Inhibits overexpressed Aurora-B kinase, suppressing NSCLC cell growth.	[142]
Lung cancer	Induces apoptosis and cell cycle arrest in S phase via receptor-mediated endocytosis.	[145]
Prostate Cancer	Enhances ROS scavenging by increasing SOD, GxP, catalase, and GSH levels.	[149]
Colorectal Cancer	Enhances 5-FU sensitivity by suppressing the miR-27a/Wnt/β-catenin pathway in CRC.	[151]
Gastric Cancer	Silences CDC20 expression, inhibiting gastric cancer cell growth.	[152]
Pancreatic cancer	Regulates oxidative and inflammatory networks, affecting immunosuppressive cytokine induction.	[153]
Liver cancer	Inhibits proliferation of liver cancer cells via apoptosis and cell cycle arrest.	[154]

**Table 3:** Summary of the anticancer properties of acacetin and its mechanisms.

Cancer Type	Findings	References
Lung cancer (NSCLC) A549 and H460	Acacetin inhibits NSCLC cell proliferation and induces apoptosis by regulating the p53/miR-34a/PD-L1 pathway.	[163]
Breast cancer T-47D and MDA-MB-231 cells	Acacetin exerts its anticancer effects on breast carcinoma cells by inducing cell cycle arrest, generating ROS, and activating RIP1-dependent necroptosis, effects that are modulated by ROS inhibition with NAC and RIP1 inhibition with Necrostatin-1.	[164]
Breast cancer MCF-7 and MDA-MB-468	Inhibition of ERK1/2 and AKT signalling and modulation of the expression of cell cycle regulators.	[164]
Colon cancer cell lines, SW480 and HCT-116	Acacetin induces mitochondrial ROS-mediated, caspase-independent cell death in SW480 and HCT-116 colon carcinoma cells through AIF activation	[166]
Prostate Cancer Cells DU145	STAT3 activity is inhibited by direct binding, leading to downregulation of its target genes.	[167]
Liver cancer cell lines HepG2	Inhibition of STAT3 activation by suppressing its phosphorylation and directly binding to STAT3, as well as inhibition of upstream kinases like c-Src, JAK1, and JAK2.	[168]
Gastric cancer MKN45 and MGC803	Acacetin suppresses the invasion, metastasis, and TGF-β1-induced EMT of gastric cancer, and the mechanism likely involves the inhibition of the PI3K/Akt/Snail signalling pathway	[161]
Ovarian cancer	Acacetin suppresses LPA production, inhibiting the RAGE-PI3K/AKT pathway and reducing cell proliferation and inflammation. However, exogenous LPA can restore this pathway and counteract acacetin’s effects.	[170]

**Table 4:** Summary of Pinocembrin and its anticancer activities.

Cancer Type	Findings	References
Breast cancer (MCF-10A cells, breast cancer cell lines MCF-7, SKBR3, and MDA-MB-231)	Inhibiting cell proliferation, inducing cell cycle arrest and apoptosis, and regulating key signalling pathways like PI3K/AKT, thereby reducing cancer cell growth and spread	[177]
Liver cancer hepatocellular carcinoma. (HepG2 and Li-7)	Pinocembrin inhibits STAT3 phosphorylation, leading to downregulation of downstream target genes, such as cyclin D1, which, in turn, suppresses cyclin E expression. This STAT3 inhibition results in reduced cell proliferation and survival	[178]
Colorectal cancer (HT29 and HCT116 cells)	Pinocembrin suppressed the proliferation, migration, invasion, and EMT of colorectal cancer cells through the regulation of LACTB	[179]
Ovarian cancer (in silico studies)	Pinocembrin induces apoptosis in ovarian cancer cells via AKT1-mTOR signalling pathway	[180]
Prostate cancer (PC-3)	Pinocembrin induces apoptosis by generating endogenous ROS and leading to G0/G1 cell cycle arrest.	[182]
Lung cancer cells (A549)	Pinocembrin suppresses the migration and invasion of NSCLC cells by inhibiting the STAT3 pathway, thereby increasing E-cadherin expression and reducing N-cadherin and vimentin levels. However, this inhibitory effect is reversed by STAT3 overexpression.	[181]

**Table 5:** Anticancer mechanism and pathway of pinobanksin and propolis.

Cancer Type	Findings	References
Bone tumour (SH-SY5Y cells)	Inhibits the proliferation of SH-SY5Y cells by interacting with BAX, Bcl-2, and CDK4/6.	[186]
Breast cancer cell lines (MCF-10A)	Significant proliferative effect against cancer cells.	[187]
Brain cancer (T98G, and LN-18)	Apoptosis induction, cell cycle arrest, and migration inhibition.	[188]
Breast cancer cells (MDA-MB-231)	Anti-proliferative, growth-inhibitory, and anti-invasive effects.	[189]
Human Tumour Cell Lines (MCF7, NCI-H460, HeLa, HepG2, MM127)	Anti-proliferative effect.	[190]

## Data Availability

No new data were created or analyzed in this study. Data sharing is not applicable to this article.

## Appendix A. List of Chemical Composition of Honey

**Component of Honey**		**References**
Water		[232,233]
Sugar	Glucose	[234]
	Fructose	[234]
	Sucrose	[234]
	Trehalose	[234]
	Isomaltose	[234]
	Maltose	[234]
	Maltulose	[234]
	Melibiose	[233]
	Rhamnose	[235]
	Maltotetraose	[234]
	Raffinose	[234]
Protein		[232]
Enzyme	Invertase	[232]
	Glucose oxidase	[236]
	Catalase	[236]
	Phosphatases	[237]
Amino acids		[233,236]
Organic acids	Gluconic acid	[235]
	Acetic acid	[233]
Lipids		[238]
Vitamins	Ascorbic acid	[236]
	Niacin	[236]
	Pyridoxine	[236]
	Thiamine	[236]
	B2 complex	[236]
	B6 pantothenic acid	[236]
Minerals		[235,236]
Carotenoids		[236]
Polyphenols	Vanillic acid	[232,235,236]
	Syringic acid	[235,236]
	Luteolin	[235,236]
	Apigenin	[235,236]
	Myricetin	[235,236]
	Ferulic acid	[239]
	Myricetin	[236,239]
	Ellagic acid	[240]
	Tricetin	[241]
	Quercetin	[232,239]
	Galangin	[236]
	Chrysin	[239,240]
	Tectochrysin	[241]
	Isorhamnetin	[240]
	Kaempferol	[232,235,239,240]
	Pinobanksin	[185]
	Pinocembrin	[236]
	Catechin	[236]
	Epicatechin	[242]
	Acacetin	[240]
	Daidzein	[243]
	Genistein	[243]
	Caffeic acid	[232,235,236,239,240]
	Chlorogenic acid	[236,240]
	Gallic acid	[232,235,236,239,240]

## Appendix B. Summary of the Anticancer Properties of Malaysian Honeys

**Honey**	**Type of Cancer Cells**	**Findings**	**In Vitro/In Vivo/Clinical Trial**	**References**
Acacia	Lung cancer cell (NCI-H460)	Inhibited cell proliferation via cell cycle arrest at G0/G1 phase, stimulation of cytokines and calcium ion, and downregulation of Bcl-2 and p53 gene	In vitro	[244]
	Human (A375) and murine (B16-F1) melanoma	Induce antiproliferative effect and hyperploid progression through G0/G1 cell cycle arrest and	In vitro	[245]
	breast cancer (MCF-7), lung cancer (A549), and colon cancer (HCT-116)	Inhibit cell proliferation with IC50 value of 5.053 μg/mL, 5.382 μg/mL, and 6.728 μg/mL against the breast, colon, and lung cancer cell lines, respectively	In vitro	[246]
	HCT116 (colon); MCF7 (breast), and HepG2 (liver)	Inhibit cell proliferation with IC50 ranged 117.99 to 482.65 µg/mL	In vitro	[247]
	Breast cancer (MCF-7)	Antiproliferative effect through induction of apoptosis.	In vitro	[248]
	Prostate cancer cell line (PC-3)	Promote apoptosis via modulation of G0/G1 phase, pro-inflammatory cytokines, calcium ions secretion and downregulation of prostate-specific antigen.	In vitro	[249]
Gelam	HCT 116 Colorectal Cancer Cells	Inhibit cell proliferation with IC50 of 75 mg/mL and induce apoptosis	In vitro	[250]
	Colon cancer (HCT 116)	Inhibit cell proliferation with IC50 2.0% *v*/*v* after 72 incubation	In vitro	[251]
	Human diploid fibroblast	Attenuated radiation-induced cell death by promoting cell cycle progression and inhibiting apoptosis by downregulating ATM, p73, and p16	In vitro	[252]
	HT 29 colon cancer	Inhibit cell proliferation with IC50 of 39.0 mg/mL by inducing apoptosis through DNA damage and suppressing inflammation via reduction in prostaglandin E2.	In vitro	[220]
	HT29 Colon cancer	Promote apoptosis via mTOR and Wnt/B-catenin pathway. -downregulated the gene expressions of Akt, mTOR, Raptor, Rictor, β-catenin, Gsk3β, Tcf4 and cyclin D1 while cytochrome C and caspase 3 genes were shown to be upregulated	In vitro	[253]
	HT29 colon cancer	Induce apoptosis by upregulating early pro-apoptotic proteins (caspase 9 and IκB) and downregulating cancer-related genes (KRAS, ERK, AKT, Bcl-xL, NFkB (p65))	In vitro	[254]
Pineapple	HT 29 colon cancer	Inhibit cell proliferation with IC50 of 85.5 mg/mL by inducing apoptosis through DNA damage and suppressing inflammation via reduction in prostaglandin E2.	In vitro	[220]
Kelulut	Cervical cancer (HeLa)	Inhibit cell proliferation with IC50 of 75.76 mg/mL)	In vitro	[255]
	Lung cancer (A549)	Inhibit cell proliferation at IC50 of 0.62% *v*/*v* by inducing cell cycle arrest at G2/M phase	In vitro	[256]
	Sprague Dawley rats	possessed chemo-preventive properties in rats induced with colorectal cancer and it was found to be not toxic to rats.	In vivo	[257]
Tualang	Sprague Dawley rats	Tualang honey ameliorated breast cancer by increasing the susceptibility of proapoptotic proteins; apoptotic protease activating factor-1 (Apaf-1) interferon-gamma (IFN-γ) interferon gamma receptor-1 (IFNGR1) tumor protein P53 (p53) and decreased the expression of anti-apoptotic proteins; tumour necrosis factor alpha (TNF-α), cyclooxygenase-2 (COX-2) and B-cell lymphoma-extra-large (Bcl-xL).	In vivo	[258]
	Sprague Dawley rats	Tualang Honey alleviated breast cancers in rats by reducing cancer cell growth and enhanced histological grading.	In vivo	[259]
	Sprague Dawley rats	Tualang honey showed chemo-preventive properties in oral squamous cell carcinoma-induced rats by suppressing cancer cell proliferation and activity and preserving cellular adhesion.	In vivo	[260]
	Sprague Dawley rats	Tualang Honey alleviated breast carcinogenesis by modulating haematologic, oestrogenic and apoptotic activities in the breast cancer animal model.	In vivo	[261]
	Breast cancer (MCF-7 & MDA-MB-231)	Tualang honey promoted apoptotic cell death induced by tamoxifen in breast cancer cell lines.	In vitro	[262]
	Breast cancer (MCF-7 & MDA-MB-231)	Tualang honey demonstrated cytotoxic and apoptotic activities against human breast cancer cell lines with the mitochondrial apoptotic pathway’s involvement. Activates caspase-3/7 and -9.	In vitro	[219]
	Breast cancer (MCF-7)	Tualang honey was found to be cytotoxic to breast cancer cell line (MCF-7) but protected non-tumorigenic epithelial breast cell line (MCF-10A) from the toxic effects of tamoxifen active metabolite 4-hydroxytamoxifen.	In vitro	[263]
	Cervical cancer (HeLa)	Tualang honey demonstrated cytotoxic and apoptotic activities against human cervical cancer cell lines with the mitochondrial apoptotic pathway’s involvement. Activates caspase-3/7 and -9.	In vitro	[219]
	Oral squamous cell carcinomas (CRL-1623)	Tualang honey showed an anti-proliferative effect on oral squamous cell carcinoma and osteosarcoma cell lines by inducing early apoptosis.	In vitro	[264]
	Human Osteosarcomas (CRL-1543)			
	Acute and chronic myeloid leukaemia (K562 and MV4-11)	Tualang honey demonstrated apoptosis-inducing ability for acute and chronic myeloid leukaemia (K562 and MV4-11) cell lines.	In vitro	[265]
	Mouse keratinocyte (PAM212)	Tualang honey protected keratinocytes from ultraviolet radiation-induced inflammation and DNA damage via modulation in early biomarkers of photocarcinogenesis.	In vitro	[266]
	Human Keloid fibroblasts (pKHDF)	The methanolic honey extraction has an antiproliferative effect on keloid fibroblasts at 50%, 25%, 12.50% and 6.25% of concentration.	In vitro	[267]
	Lung cancer (A549 and H23)	TH inhibited the proliferation of ADC cells in a dose- and time-dependent manner through cell cycle arrest at the G2/M phase. TH induces apoptosis through both intrinsic and extrinsic pathways.	In vitro	[268]
	Lung cancer (A549 and H23)	TH exerts anticancer activity through modulation of pertinent cancer-related proteins associated with cell proliferation, apoptosis, and angiogenesis, such as HRAS, HSPB1, TUBA1C, PCNA, ELAVL1, and H3F3A.	In vitro	[269]
	Lung cancer (A549)	TH promote apoptosis of A549 cells through alteration of the PI3K/AKT signaling pathway-related proteins.	In vitro	[270]
	Head and neck cancer	Tualang honey improved cancer-related fatigue and quality of life of patients with head and neck cancer post-chemotherapy or radiotherapy.	Human study	[271]
	Breast cancer	Combination of Tualang honey and anastrozole showed more improvement in decreasing breast background parenchymal enhancement in patients with breast cancer than anastrozole alone.	Human study	[272]
